# Spray Pyrolyzed TiO_2_ Embedded Multi-Layer Front Contact Design for High-Efficiency Perovskite Solar Cells

**DOI:** 10.1007/s40820-020-00559-2

**Published:** 2021-01-04

**Authors:** Md. Shahiduzzaman, Mohammad Ismail Hossain, Sem Visal, Tetsuya Kaneko, Wayesh Qarony, Shinjiro Umezu, Koji Tomita, Satoru Iwamori, Dietmar Knipp, Yuen Hong Tsang, Md. Akhtaruzzaman, Jean-Michel Nunzi, Tetsuya Taima, Masao Isomura

**Affiliations:** 1 Nanomaterials Research Institute (NanoMaRi), Kanazawa University, Kakuma, Kanazawa, 920-1192 Japan; 2 Department of Applied Physics and Materials Research Center, The Hong Kong Polytechnic University, Hung Hom, Kowloon, Hong Kong, P. R. China; 3 Research Institute of Science and Technology, Tokai University, Kitakaname, Hiratsuka, 259-1292 Japan; 4 Department of Chemistry, School of Science, Tokai University, Kitakaname, Hiratsuka, 259-1292 Japan; 5 Graduate School of Engineering, Tokai University, Kitakaname, Hiratsuka, 259-1292 Japan; 6 Department of Modern Mechanical Engineering, Waseda University, 3-4-1 Okubo, Shinjuku, Tokyo, 169-8555 Japan; 7 Solar Energy Research Institute, The National University of Malaysia, 43600 Bangi, Selangor Malaysia; 8 Department of Materials Science and Engineering, City University of Hong Kong, Kowloon, Hong Kong, P. R. China; 9 Geballe Laboratory for Advanced Materials, Department of Materials Science and Engineering, Stanford University, Stanford, USA; 10 Department of Physics, Engineering Physics and Astronomy, Queens University, Kingston, ON Canada

**Keywords:** Perovskite, Tandem solar cells, Spray pyrolysis deposition, TiO_2_ compact layer, Optics and optimization, Electrical characteristic

## Abstract

The photovoltaic performance of perovskite solar cells (PSCs) can be improved by utilizing efficient front contact. However, it has always been a significant challenge for fabricating high-quality, scalable, controllable, and cost-effective front contact. This study proposes a realistic multi-layer front contact design to realize efficient single-junction PSCs and perovskite/perovskite tandem solar cells (TSCs). As a critical part of the front contact, we prepared a highly compact titanium oxide (TiO_2_) film by industrially viable Spray Pyrolysis Deposition (SPD), which acts as a potential electron transport layer (ETL) for the fabrication of PSCs. Optimization and reproducibility of the TiO_2_ ETL were discreetly investigated while fabricating a set of planar PSCs. As the front contact has a significant influence on the optoelectronic properties of PSCs, hence, we investigated the optics and electrical effects of PSCs by three-dimensional (3D) finite-difference time-domain (FDTD) and finite element method (FEM) rigorous simulations. The investigation allows us to compare experimental results with the outcome from simulations. Furthermore, an optimized single-junction PSC is designed to enhance the energy conversion efficiency (ECE) by > 30% compared to the planar reference PSC. Finally, the study has been progressed to the realization of all-perovskite TSC that can reach the ECE, exceeding 30%. Detailed guidance for the completion of high-performance PSCs is provided.

**Electronic supplementary material:**

The online version of this article (10.1007/s40820-020-00559-2) contains supplementary material, which is available to authorised users.

## Highlights

- Industrially viable bottom-up spray pyrolysis deposition technique was used to prepare the highly compact TiO_2_ film, which is a vital element for the multi-layer front contact.
- The optimization of the front contact is presented by fabricating reproducible and efficient perovskite solar cells
- Multi-layer front contact is applied to realize efficient perovskite single-junction and perovskite/perovskite tandem solar cells, where optics and electrical effects of solar cells are studied by optically coupled 3D electromagnetic simulations.

**Electronic supplementary material:**

The online version of this article (10.1007/s40820-020-00559-2) contains supplementary material, which is available to authorised users.

## Graphical abstract

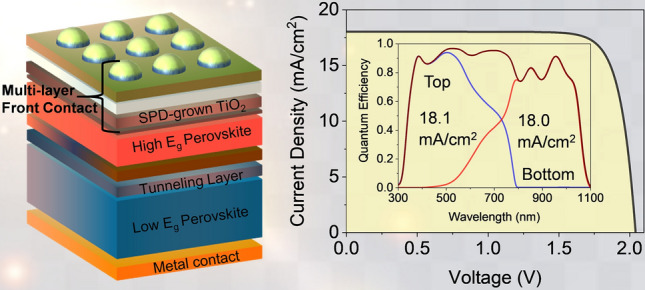

## Introduction

Perovskite material systems have attracted considerable research attention to the academic and industrial communities due to their exciting optoelectronic properties, including tunable bandgap, high absorption coefficient, more considerable diffusion length, and low processing costs [1–5]. The energy conversion efficiency (ECE) of single-junction perovskite solar cells (PSCs) has now increased rapidly by more than 25% [6], since Miyasaka and co-workers first reported perovskites as a photo-absorber material with an ECE of 3.8% [3]. Furthermore, the multi-bandgap property of perovskites allows realizing high-efficiency tandem solar cells (TSCs) [5, 7–11], which have a tremendous ability to surpass the Shockley-Queisser (SQ) limit of silicon solar cells [12, 13]. According to the detailed-balance theory, ECE of the TSC may go beyond 45% if the optimum material bandgaps (~ 1.1 and ~ 1.73 eV) are selected [9, 12]. However, high-efficiency PSCs reported in literature often suffer from a reproducibility problem, which is one of the significant issues in PSC fabrication and their future commercialization [14]. Typically, the front contact has a significant influence on the electrical and optical properties of a PSC, which needs to be efficient enough so that it can fulfill some fundamental requirements (e.g., improved light incouping, high optical transparency, high lateral conductivity, and low absorption loss) for achieving efficient photon absorption in the solar cell [15]. The front contact with a single layer cannot facility all essential benefits; hence, it requires a multi-layer architecture for efficient PSCs. In a multi-layer front contact, a hole blocking/electron transport layer (ETL) is considered a key element that needs to be compact, smoothly distributed, and pin-hole-free to achieve high ECEs [16–18]. In addition, the qualities of the ETL, such as energy level alignment, charge mobility, morphology, and related interface properties, are prominently valuable for the determination of PSCs with the better photovoltaic performance [15, 19]. Hence, a detailed understanding of the front contact design, deposition techniques, and optoelectronic properties of materials are essential while aiming for high-efficiency PSCs.

The current study preliminary focuses on the selection, preparation, and optimization of the ETL so that the optimized ETL can be utilized to make efficient multi-layer front contact for realizing PSCs with high-efficiency. Until now, a wide variety of metal oxides, such as ZnO, SnO_2_, TiO_2_, and WO_3_, have been investigated as electron transport materials (ETMs) ought to their high electron mobility and enhanced environmental stability [8, 15, 20–22]. Amongst, TiO_2_ is considerably popular due to its potential simple deposition, suitable energy level, and tunable electronic properties [23–25]. Furthermore, TiO_2_ can produce a smooth film surface to improve charge transfer while maintaining the uniformity [19, 23]. Several deposition methods, such as spin-coating, spray pyrolysis deposition (SPD), atomic layer deposition (ALD), chemical vapor deposition (CVD), sputtering, have been used to prepare TiO_2_ compact layer (CL) [18, 19, 23, 26–30]. The current work investigates the preparation of high-quality (compact, uniform, and reproducible) TiO_2_ films by the SPD technique so that the film can be used as an ETL for the fabrication of efficient PSCs. The SPD technique is commonly used in industries for a massive area film deposition [31]. The study also focuses on the optimization and reproducibility of the film while fabricating the real devices. Next, the study progressed to the realization of the multi-layer front contact embedded with an optimized TiO_2_ CL, so that the efficiency of both single-junction PSCs and perovskite/perovskite TSCs can be maximized. A comparison of the investigated outcome with the theoretical upper limit of PSCs will be provided. The optics and optimization processes are studied by three-dimensional finite-difference time-domain (FDTD) simulations. The electrical characteristics of solar cells are investigated through the finite element method (FEM) simulations. A fair comparison between experimental results and simulation findings will be provided to validate the numerical approach.

## Methods

### Materials and Characterization

Lead iodide (PbI_2_) and methylammonium iodide (CH_3_NH_3_I) were purchased from Tokyo Chemical Industry (Tokyo, Japan). Titanium diisopropoxide bis (acetylacetonate; Aldrich, 75 wt%) in isopropanol was bought from the Wako chemical. *N,N*-dimethylformamide (DMF, purity 99.5%) and Dimethyl sulfoxide (DMSO, purity 99.5%) were also purchased from Wako Chemical (Tokyo, Japan). The field emission scanning electron microscopy (FE-SEM; S-4800, Hitachi High-Tech, Tokyo, Japan) was used to analyze the resulting surface morphologies. The ultraviolet–visible near-infrared spectrophotometer (UV–Vis-NIR; V-670, Jasco Corporation, Tokyo, Japan) was used to measure the absorption spectra from the deposited samples. The X-ray diffraction (XRD) patterns of perovskite films were measured using an X-ray diffractometer (D8 Discover, Bruker AXS Co. Ltd, Tokyo, Japan) with an X-ray tube (Cu K*α* radiation, *λ* = 1.5406 Å). The current density *versus* voltage (*J-V*) characteristics at a scan speed of 0.05 V s^–1^ with forward scan (FS; from −0.1 to 1.2 V) and reverse scan (RS; from 1.2 V to −0.1 V) of the resultant devices was analyzed under simulated (100 mW cm^−2^, AM1.5, 1 sun intensity) by a solar simulator using a Keithley 2401 digital source meter. The incident photon-to-electron conversion efficiency (IECE) of the resultant devices was tested using a monochromatic xenon arc light system (Bunkoukeiki, SMI-250JA). All the devices were characterized in air, humidity ranging from 40 to 50%, and temperature around 22 °C. The active area of the device was 0.09 cm^2^.

### Device Fabrication

The transparent conducting fluorine-doped tin oxide FTO/patterned glass substrates with a sheet resistance of 10 Ω sq^−1^ were cleaned with soap solution, distilled water, acetone, ethyl alcohol, and again distilled water. Subsequently, substrates were further cleaned by UV-ozone treatment for 15 min. Then, a compact TiO_2_ layer was deposited on the FTO glass via SPD at 450 °C from a precursor solution of titanium diisopropoxide bis(acetylacetonate) in isopropanol according to the procedure described by Wakamiya et al. [32]. As deposited substrates were left at 450 °C for 30 min in a muffle furnace and let to cool down to room temperature. The perovskite precursor solution (1 M PbI_2_ and 1 M CH_3_NH_3_I in DMF and DMSO mixed solvent) was coated on the resulting substrates. A mixed solution of 1 M PbI_2_ and 1 M CH_3_NH_3_I was dissolved in a mixed solvent of DMF and DMSO (4 V DMF: 1 V DMSO), followed by at 60 °C for 1 h. The perovskite film was prepared by spin-coating the precursor solution at 6000 rpm for 60 s with dripping 500 µL of chlorobenzene just 8 s after the spin-coating started. The precursor coated substrates were then annealed at 100 °C on a hot plate for 1 h to crystallize perovskite in a glove box under an inert environment. We tuned the thickness of TiO_2_ CL by changing the concentration (0.15, 0.20, 0.25, 0.30, 0.35, and 0.40 M) of TiO_2_ solution. The champion cell has a TiO_2_ thickness of 70 nm with the precursor solution concentration of 0.35 M. The thickness of the perovskite absorber was measured to be approx. 300 nm. It is noted that the Perovskite layer only contributes to quantum efficiency (*QE*) and short-circuit current density (*J*_*SC*_). The detailed hole transport layer (HTL) precursor solution preparation is reported elsewhere [32]. Deposited Spiro-OMeTAD HTL has a thickness of 250 nm. Finally, 100-nm-thick gold (Au) electrodes were deposited on top of the HTL layer to complete the device fabrication.

### Coupled Opto-electrical Simulation Method

The Finite-difference time-domain (FDTD) approach was carried out to investigate the optical wave propagation in single-junction PSCs and perovskite/perovskite TSCs in three-dimension (3D), where precise electromagnetic calculations are solved by Maxwell’s equations. The FDTD is one of the most suitable techniques for examining the optics of several optoelectronic devices due to its accurate estimation and broadband frequency coverage. Experimentally realized complex refractive indices were used as input parameters for the optical simulations, which are provided in Fig. S15. Most optical constants were determined from the ellipsometry measurement of deposited films; however, the refractive index and extinction coefficient of MASnPbI_3_ and SnO_2_ materials were adapted from the literature [33, 34]. A circularly polarized incident plane wave was considered as a source with the standard AM 1.5G solar spectrum, which has an amplitude of 1000 V m^−1^. The source was placed in the air that propagates to the solar cell structure from front contact to the back contact. The monochromatic source wavelengths were selected based on the bandgap of the absorber materials. In the current study, optical simulations were carried from 300 to 800 nm for single-junction PSCs, and 300 to 1100 nm was chosen for perovskite/perovskite TSCs. Furthermore, necessary boundary conditions were applied to the simulation region, where the perfect matched layer (PML) boundary condition (BC) was selected for the z-directional outgoing waves so that unwanted reflections can be evaded. In the x- and y- direction, periodic BCs were applied so that periodicity can occur throughout the solar cell structure. The convergence test was performed to attain the accurate mesh size, where the dimension of mesh grids was 5 nm in all cases. Moreover, the layer thickness optimization was carried out by the particle swarm optimization (PSO) embedded with the FDTD method, which allows finding the optimum parameter so that photon absorption in the perovskite layer is maximized, and optical losses are minimized. The PSO algorithm is even much effective while studying a complex geometrical structure. By considering the valid assumption, where each incident photon generates an electron–hole pair (EHP) if the bandgap of the perovskite absorber is lower than the incident photon energy. A further detail on how to calculate total generation rate, power densities, *QE*, *J*_SC_, and *V*_OC_ are provided in Sect. S2.

To estimate the realistic photovoltaic performance, electrical parameters play an additional critical role. However, optical simulations do not allow determining electrical parameters of the solar cell. Therefore, as a mandatory part, electrical simulations were performed thoroughly of investigated solar cells by the finite element method (FEM), which allows determining the current–voltage (*J-V*) characteristic curve of the solar cell. A set of necessary electronic parameters of materials was used for the calculation, which were adapted from the literature and summarized in Table S5 [35–40]. A more detailed description of the electrical simulation is provided in Section S3 and literatures [11, 37]. Such an advanced numerical approach with the combination of optical and electrical simulations provides excellent agreement with the experiments [36, 37, 41], as experimentally realized material properties were used in the simulation environment.

## Results and Discussion

### Planar Solar Cell Design and Film Preparation

**Fig. 1:**
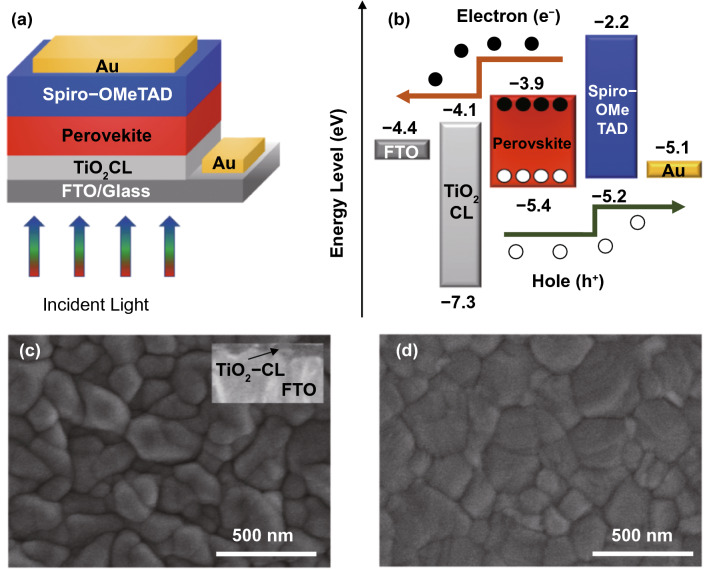
**a** Schematic diagram and **b** corresponding energy levels of the investigated single-junction planar perovskite solar cell. Top-view SEM micrographs of **c** spray pyrolysis deposited TiO_2_ compact layer (the inset shows the cross-sectional view of the TiO_2_), and **d** perovskite film deposited on TiO_2_-CL/FTO substrate. The TiO_2_ precursor solution has a concentration of 0.35 M and a thickness of 70 nm

In the first step of this study, a planar PSC was investigated. Approximately 300-nm-thick methylammonium lead iodide (MAPbI_3_) perovskite with a bandgap of ~ 1.5 eV is placed between charge transport layers to form the n-i-p junction. A schematic diagram of the planar PSC is depicted in Fig. 1a**,** and corresponding energy levels are shown in Fig. 1b [42, 43].

As TiO_2_-CL is one of the key elements for PSCs; hence, the TiO_2_ film's optimization has been carried out to determine the optimum ETL thickness. The TiO_2_ film was prepared on the FTO substrate, where the TiO_2_ precursor solution was tuned from 0.15 to 0.40 M. It has been discovered that the TiO_2_-CL with a precursor concentration of 0.35 M, with a thickness of ~ 70 nm, only exhibits excellent uniformity and smooth surface morphology, as shown in Fig. 1c. Fig. 1d illustrates the top-view SEM image of MAPbI_3_ film deposited on the TiO_2_/FTO, which shows a standard surface morphology with densely packed perovskite crystals where grain size varies approximately from 200 to 500 nm. Figure S1a shows the UV–vis absorption spectrum for the wavelength range of 300–820 nm. The absorbance goes zero while reaching the wavelength of 800 nm, which proves the perfection of perovskite material properties. The XRD patterns are illustrated in Fig. S1b, where diffraction peaks are detected at 2θ angles of 14.1°, 28.7°, 31.8°, and 40.74° in TiO_2_ CL/MAPbI_3_ film, which corresponds to (110), (220), (310), and (224) crystal planes, respectively. There was no peak from PbI_2_ at 12.6°, implying that the complete transformation of PbI_2_.

### Reproducibility of Single-junction Planar Perovskite Solar Cells

**Fig. 2:**
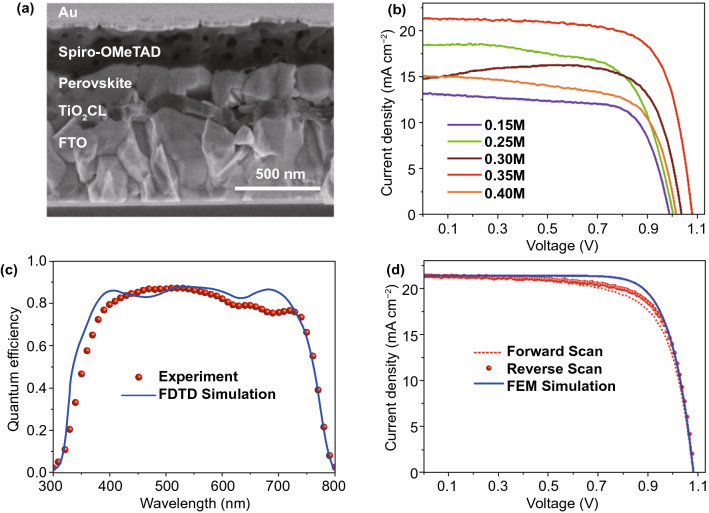
**a** Cross-sectional FESEM image of the fabricated champion planar perovskite solar cell. **b** Current–voltage (*J-V*) curves with the reverse scan (RS) for the fabricated planar PSCs with different TiO_2_ precursor solution concentration. **c** Comparison of quantum efficiencies between experiment and FDTD optical simulation. **d** Forward scan (FS) and reverse scan (RS) *J-V* curves of the fabricated best performing PSC along with the *J-V* curve realized from the FEM electrical simulation

The effect of the TiO_2_ hole blocking layer on the photovoltaic performance of single-junction planar PSCs is studied. A PSCs group with the structure FTO/TiO_2_ CL/MAPbI_3_/Spiro-OMeTAD/Au was fabricated, where the device performance was analyzed under one sun (AM 1.5G, 100 mW cm^–2^) conditions. The concentration of TiO_2_ precursor solution was varied from 0.15 to 0.40 M while fabricating the device. A cross-sectional SEM image of the fabricated planar PSC is depicted in Fig. 2a, where a TiO_2_ CL is placed between the MAPbI_3_ absorber and FTO glass substrate. The typical current–voltage (*J-V*) characteristic curves with the reverse scan (RS) of the fabricated PSCs against various concentrations of TiO_2_ precursor solution are presented in Fig. 2b. The *J-V* curves of fabricated PSCs with forward scan (FS) and reverse scan (RS) are given in the Supporting Information (Fig. S2). A wide discrepancy of *J-V* curves is observed while varying the concentration of the TiO_2_ precursor solution. The photovoltaic performance of fabricated devices with different TiO_2_ precursor solution concentrations was compared using photovoltaic parameters extracted from *J-V* curves, as shown in the supporting information (Fig. S3). A variation of the TiO_2_ precursor solution concentration leads to *J*_SC_, *V*_OC_, and *FF* from 13.3 to 21.3 mA cm^−2^, 0.99 to 1.08 V, and 65.8% to 72%, respectively, with the RS.

On the other hand, *J*_SC_, *V*_OC_, and *FF* ranged from 15.07 to 21.4 mA cm^−2^, 0.99 to 1.075 V, and 50.4% to 69%, respectively, with the FS. *J*_SC_ and *V*_OC_ are only maximized in the case of 0.35 M, where the TiO_2_ CL has a 70-nm thickness, resulting in improved ECE. As compared to others, the 0.35 M condition exhibits a low hysteresis problem, allowing it to reach an ECE of 16.55%. In addition to that, light soaking and moisture stability tests of fabricated planar PSCs are provided in Fig. S4. More detailed information on such devices' stability can be found from our previously published works in the literature [26, 44]. Up to our knowledge, this is the uppermost ECE, which is experimentally realized from the basic planar PSC with the incorporation of only the SPD-grown TiO_2_ CL as the ETL. Further optimization of planar PSCs has been carried by utilizing 3D-FDTD optical simulation integrated with particle swarm optimization (PSO) algorithm to determine the thickness of the TiO_2_ CL. A detailed description of the optical simulation is provided in the methods section. It has been found that the thickness of a TiO_2_ CL in PSCs has a significant impact on the optics of the solar cell. The TiO_2_ CL thickness dependent QEs and parasitic absorptions are presented in Fig. S5. The optical simulation findings provided an excellent agreement with the experimentally realized outcome, where the QE of a planar PSC is maximized by reducing parasitic loss only when the TiO_2_ CL has a thickness of 70 nm. A comparison of experimentally realized and simulated QEs is shown in Fig. 2c. The TiO_2_ ETL absorbs a major portion of incident photons in the UV range; however, photons within the visible wavelength range (400–700 nm) are primarily absorbed by the perovskite absorber layer. Noticeably, both QEs exhibit almost identical characteristics after 750 nm wavelength, which shows the work’s validity. Such optical phenomena in the solar cell can be further verified by their calculated power density and electrical distribution profiles given in Figs. S6, S7. By looking at power densities and electric distributions, the QE is thought to be understood only from constructive and destructive waves due to forward and backward wave propagations. No scattering or diffraction is noticeable in the planar configuration of the PSC, which also suggests that a significant portion of incident light is lost only due to high reflections. Such reflections can be minimized, and photon absorption can be enhanced in the PSC using multi-layer photonic structures [45]. The PSC integrated with a front photonic structure can further reduce the UV radiation, improving device stability [25, 45].

However, optical simulations do not provide electrical parameter values for the solar cell; hence, FEM simulations were adapted to investigate the electrical effects of the solar cell by allowing a *J-V* characteristic curve. A wide range of essential electronic properties of materials was used for the FEM calculations. More details on the FEM electrical simulations are provided in the Methods section. A comparison of *J-V* characteristic curves between experiment and simulation is provided in Fig. 2d for the best performing device. The *J-V* curves show a comparable characteristic by validating the experimental data with numerical simulations. Corresponding extracted photovoltaic parameters from the *J-V* curves are summarized in Table 1.

**Table 1:** A comparison between experiment and simulation for photovoltaic performance parameters extracted from *J-V* characteristics of planar PSCs, as shown in Fig. 2d

Method	Performance Parameters			
	*V*_oc_ (V)	*J*_sc_ (mA cm^−2^)	FF (%)	ECE (%)
Experiment (FS)	1.07	21.48	65	15.16
Experiment (RS)	1.07	21.30	72	16.55
FEM Simulation	1.07	21.40	76	17.47

The *J*_SC_ and the *V*_OC_ are almost equivalent in each case; however, the fabricated device's ECE (16.55%) is limited due to a slight reduction of the *FF* compared to the simulated device (ECE ~ 17.47%). It is assumed that the imperfection of interfaces between perovskite and contact layers may lead to a lower *FF* in the real fabrication.

**Fig. 3:**
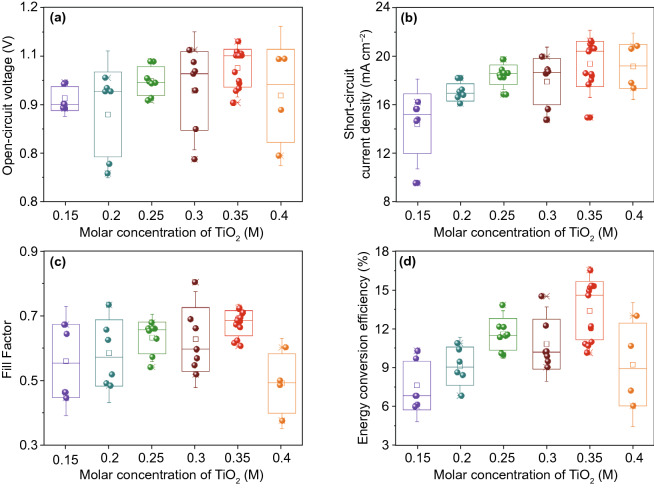
Average values of **a** open-circuit voltage (*V*_OC_), **b** short-circuit current density (*J*_SC_), **c** fill-factor (*FF*), and **d** energy conversion efficiency (*ECE*) obtained from resultant 13 fabricated devices in each group while varying the molar concentration of the TiO_2_ precursor solution from 0.15 to 0.40 M

As the next step of this study, we started investigating PSC reproducibility by considering 13 fabricated devices in each group (total 6 groups) to attain an optimized planar PSC design for future investigations. As before, the concentration of the TiO_2_ precursor solution was ranged from 0.15 to 0.40 M while fabricating the PSC device. The photovoltaic parameters were extracted from each *J-V* curve. The average values of *V*_OC_, *J*_SC_, *FF*, and ECE of resultant fabricated PSCs as a function of TiO_2_ precursor solution concentration are illustrated in Fig. 3a-d. Error bars indicate plus or minus the standard deviation from the mean value.

As anticipated, PSCs with the 0.35 M TiO_2_ precursor solution concentration demonstrated a superior performance as compared to other cases (0.15, 0.20, 0.25, 0.30, and 0.40 M). The 0.35 M case yield better reproducibility and average *V*_OC_, *J*_SC_, *FF*, ECE of 1.02 ± 0.06 V, 19.37 ± 1.9 mA cm^−2^, 67 ± 5%, 13.5 ± 3.05%, respectively. The statistics of PSC performances of 13 devices are summarized in Table S1, where results can be attributed to the enhanced morphology and crystallinity of perovskite films using optimized TiO_2_ precursor solution concentration. The best-performance PSC has an optimized TiO_2_ ETL thickness of approx. 70 nm, which shows a maximum ECE of 16.55% with a *J*_SC_ of 21.30 mA cm^−2^, a V_OC_ of 1.07 V, and a FF of 72%. However, as the photovoltaic performance of planar PSCs yet limited due to higher optical losses, next part of this study focuses on the efficient photon management so that light incoupling and/or light trapping in the solar cell can be improved.

### Photon Management in Perovskite Solar Cells

The fabricated basic planar PSC with the TiO_2_-CL ETL has the potential to reach high ECE. However, the present device design does not allow adequate photon absorptions in the perovskite absorber because of higher optical losses that prevents the realization of efficient PSCs. Efficient photon management allows realizing PSCs with high ECEs with the improved light incoupling and/or light trapping. By texturing the solar cell interfaces, improved light incoupling and light trapping in the solar cell can be achieved; however, this negatively influences the electrical parameters, such as *V*_OC_ and *FF*. Efficient solar cell mostly requires flat interfaces between absorbers and contacts to ensure better electrical parameters. Nevertheless, a single compact layer of TiO_2_ is not enough for efficient electron extraction/hole blocking from the perovskite absorber, which also suffers from severe recombination problems at the perovskite/front contact interface that limits the electrical performance of the solar cell. Hence, incorporation of mesoporous TiO_2_ (mp-TiO_2_) could be one of the potential solutions which contributes to the effective electron transportation. The mp-TiO_2_ nanoparticles further allow reducing the recombination of carriers. In addition to that, the mp-TiO_2_ contributes to the realization of dense and pinhole-free perovskite film, resulting in the higher crystallinity. Subsequently, the electrical parameter (*V*_*OC*_*, FF*) values are improved. The mp-TiO_2_ additionally contributes to the enhancement of photon absorptions through the photon scattering in the active layer, leading to achieve high *J*_*SC*_ and ECE.

**Fig. 4:**
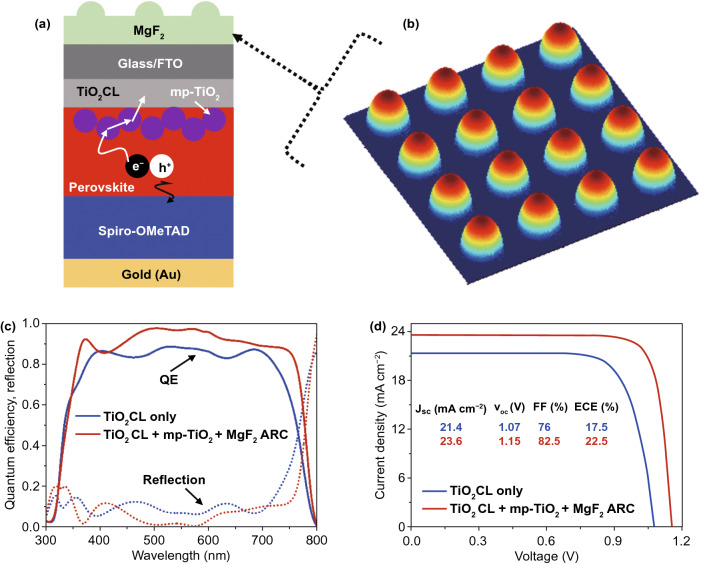
**a** Schematic diagram of the optimized single-junction perovskite solar cell. **b** Graphical representation of the nanostructured MgF_2_ ARC layer. A comparison of **c** quantum efficiencies and corresponding reflections, and **d** *J-V* characteristic curves between optimized PSC (MgF_2_ ARC/FTO/TiO_2_/mp-TiO_2_/MAPbI_3_/Spiro/Au) and planar reference PSC (FTO/TiO_2_/MAPbI_3_/Spiro/Au)

In the optimized device design, we have used a multi-layer front contact by the use of metal oxides, which has a comparable refractive index (2.2 ~ 2.5) with the perovskite absorber. Thus, the layer stack consisting of front contact and perovskite absorber can be treated as a single block, which leads to reduce reflections at the front contact/perovskite interface and helps to maximize *QE* and *J*_*SC*_ [46, 47]. Consequently, the optical design of the solar cell is simplified, and by only focusing the light incoupling, photon absorptions can be improved, and reflections can be minimized. Light incoupling can be further enhanced by using a textured anti-reflection (ARC) layer, which has a refractive index of around ~ 1.4. Such an ARC layer acts as a refractive index gradient while incident light propagates from air to the solar cell. In this study, efficient photon management was achieved by improving light incoupling and light trapping. The light incoupling was attained by the utilization of a distinctive periodic semi-sphere shaped MgF_2_ layer, which facilitates like a broadband ARC so that light incoupling can be significantly improved by minimizing reflections. A part of light trapping was achieved by introducing mp-TiO_2_ nanoparticles. A schematic diagram of the proposed PSC structure by incorporating a multi-layer front contact layer is depicted in Fig. 4a. Figure 4b shows the graphical representation of the MgF_2_ ARC layer, which was deposited on top of the solar cell. The semi-sphere nanostructure can be prepared by casting an MgF_2_ film on a crystalline silicon master, which is patterned by silicon semiconductor processing [48]. The ARC is characterized by a circular base arranged nanostructure in a square grid, which can provide almost ideal light incoupling in the solar cell. The diameter of the nanostructure is assumed to be the same as the diameter of mp-TiO_2_ nanoparticle; in this study, it was 100 nm. We have integrated the ARC nanostructure in a PSC, as shown in Fig. 4a, where all solar cell interfaces are assumed to be flat. Such ARC nanostructure and mp-TiO_2_ nanoparticle allow realizing an improved photon absorption in the absorber layer, which can be seen from the QE plot illustrated in Fig. 4c.

**Fig. 5:**
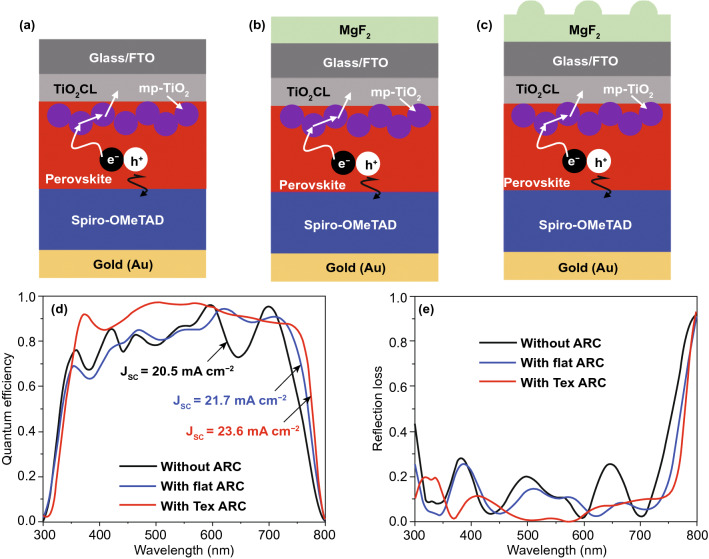
Schematic diagram of the single-junction perovskite solar cell (PSC) **a** without MgF_2_ ARC layer, **b** with a flat MgF_2_ ARC layer, and **c** with a textured MgF_2_ ARC layer. A comparison of **d** quantum efficiencies (*QE*s) and Short-circuit current densities (*J*_*SC*_s), and **e** corresponding reflection losses. The perovskite absorber has a thickness of 300 nm

In addition to this, the *QE* of the investigated planar PSC is included in Fig. 4c. A further comparison of *QE*s, *J*_*SC*_s, and reflection losses of PSCs without ARC, with a flat ARC, and with a nanostructured ARC is provided in Fig. 5. It has been observed that only the incorporation mp-TiO_2_ nanoparticles is not sufficient to improve the photon absorption in the perovskite layer, somewhat decreasing the *QE* and *J*_*SC*_ compared to the reference planar PSC is due to higher optical losses. A significant enhancement in the *QE* is realized only from the optimized structure due to reducing the optical losses. The considerable portion of optical losses is diminished owing to the minimum reflection losses.

A comparison of *QE*s and reflection losses between optimized and other structures are illustrated in Figs. 4c and 5d, e. Throughout the spectrum, the *QE* of the optimized PSC is improved compared to the planar reference PSC. The *QE* is maximized when the front ARC provides efficient light incoupling and 2 × *d* ≥ *t*_p_, where d is the absorber thickness, and *t*_p_ is the penetration depth of the absorber. On the other hand, if *t*_p_ ≥ 2 × *d*, *QE* is maximized due to light trapping. In this case, a 300-nm-thick perovskite absorber layer was considered in the solar cell structure. Hence, the *QE* enhancement for the short wavelengths up to 660 nm, mostly because of almost perfect light incoupling, whereas at wavelength over 660 nm, *QE* improvement is found due to enhanced light scattering and trapping of the incident light. At 500 nm, the *QE* of the optimized PSC reaches a peak (> 95%), which leads to a corresponding reflection of almost 0. Moreover, a reduced *QE* is observed between 370 to 470 nm, wavelength range, which is due to unwanted reflections at the front of the solar cell. However, the *QE* drops for the longer wavelengths due to non-radiative optical losses caused by back contact. Such optical phenomena can be further appropriately understood by power densities and electric field distributions. Figures S8 and S9 show power density and electric field distribution profiles of the optimized single-junction PSC under for an incident wavelength ranging from 300 to 800 nm. It is clearly observed that how the ARC layer contributes to the enhanced light incoupling, and the mp-TiO_2_ nanoparticles provide photon absorption enhancement through the scattering of the incident light, leading to the formation of a standing wave in the solar cell. Thus, it is confirmed that both nanostructured ARC and mp-TiO_2_ exhibit almost perfect light incoupling and light trapping, which leads to higher *QE* and greater *J*_*SC*_ in the simulated spectral range. In comparison to the reference planar PSC, the *J*_SC_ of the optimized PSC is increased from 21.4 to 23.6 mA cm^−2^ because of enhanced photon absorption and minimized optical losses. Such heightening in the light generation additionally also improves the electrical performance parameters of the solar cell. Fig. 4d shows the comparison of investigated J-V characteristics between reference planar and optimized single-junction PSCs. Corresponding extracted photovoltaic performance parameters are listed in Table 2.

**Table 2:** A comparison of photovoltaic performance parameters of single-junction PSCs extracted from *J-V* characteristic curves

Structure type	Performance Parameters			
	*V*_oc_ (V)	*J*_sc_ (mA cm^−2^)	FF (%)	ECE (%)
FTO/**TiO**_**2**_** CL** /MAPbI_3_/Spiro/Au	1.07	21.4	76	17.47
**MgF**_**2**_/FTO / **TiO**_**2**_** CL/ mp-TiO**_**2**_/ MAPbI_3_/Spiro/Au	1.15	23.6	82.5	22.5

A noticeable improvement of the photovoltaic parameters was found in the case of the optimized PSC, where the *V*_*OC*_ and *FF* were improved by 7.5% and 8.6%, respectively, as compared to the reference planar PSC, leading to the enhancement of ECE from 17.47% to 22.5%. As compared to the fabricated planar PSC, the ECE of optimized PSC is improved by ~ 36%. Besides, the influence of electronic parameters, mainly, dopant concentrations on the solar cell performance is provided in Fig. S10 and Tables S2-S4. Since the optimized multi-layer front contact has the potential for realizing high-efficiency single-junction PSCs, the study has been progressed to the investigation of a Perovskite/perovskite TSC for realizing high photovoltaic performance. A detailed guideline for achieving efficient Perovskite/perovskite TSCs is provided in the following section.

### Realization of High-efficiency Perovskite/Perovskite Tandem Solar Cells

In general, the design of efficient TSC is challenging because the current from the top to bottom cell in the TSC must be matched under short-circuit current conditions. Furthermore, the bandgap of the bottom cell absorber in a TSC has to be low so that a high *J*_SC_ can be reached, which can further contribute to maximizing the matched J_SC_ and ECE of the TSC. Hence, in this study, the investigation originated from the optimization of narrow bandgap single-junction PSCs. The upper limit of the *J*_SC_ of the perovskite/perovskite TSC can be predicted from the bottom cell *J*_SC_, which is 50% of the bottom cell *J*_SC_. It is assumed that the low bandgap perovskite can exhibit high *J*_*SC*_, where several studies reported remarkable ways to lower the bandgap by using the Pb/Sn binary perovskite (MAPb1_-x_Sn_x_I_3_) alloys [49–51].

**Fig. 6:**
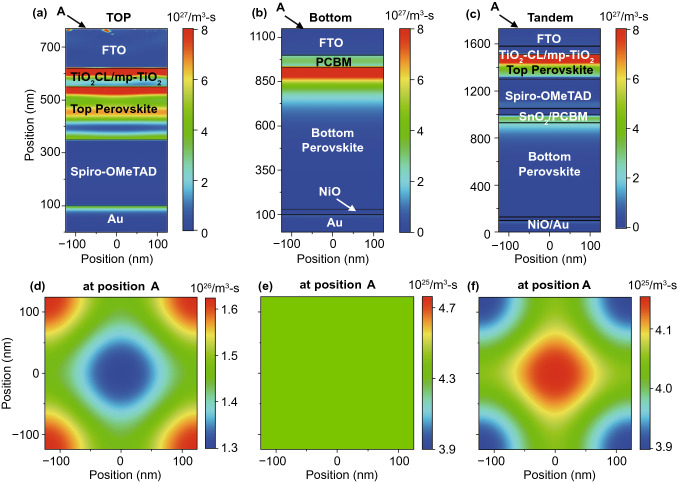
Total generation rate of **a** single-junction top perovskite (wide *E*_g_) solar cell, **b** single-junction bottom perovskite (narrow *E*_g_) solar cell, and **c** perovskite/perovskite tandem solar cell. **d-f** Corresponding top-view of the generation rate. The top perovskite absorber has a thickness of 210 nm, and the bottom perovskite absorber has a thickness of 800 nm

**Fig. 7:**
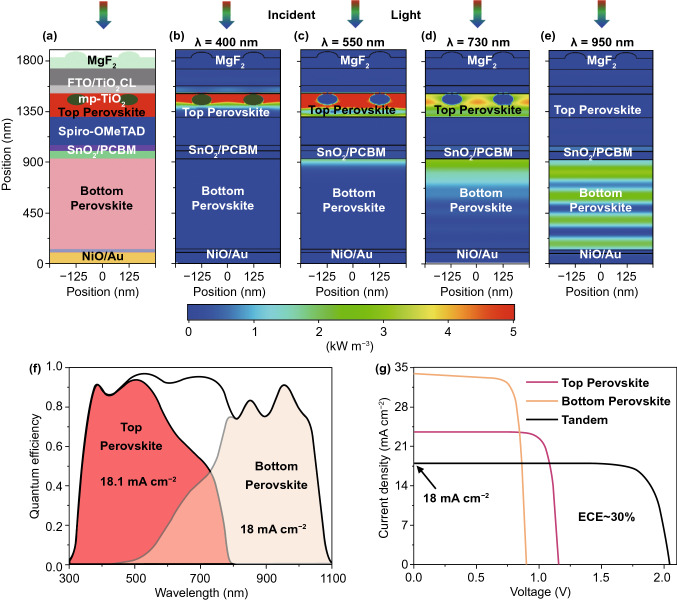
**a** Schematic cross-section of the optimized perovskite/perovskite tandem solar cell. Corresponding power density profile for an incident wavelength of **b** 400 nm, **c** 550 nm, **d** 730 nm, and **e** 950 nm. **f** Quantum efficiency and **g** current–voltage characteristic curves of the best-investigated perovskite/perovskite tandem solar cells under matching short-circuit current condition, where top perovskite and bottom perovskite has a thickness of 210 and 800 nm, respectively.

In this study, we propose the use of MASnPbl_3_ perovskite (*E*_g_ ~ 1.16 eV) absorber, which is sandwiched between PCBM and NiO charge transporting layers. NiO provides excellent chemical stability with a suitable work function, exhibiting a higher *V*_OC_ of the PSC [52]. The absorber thickness plays an essential role in maximizing *QE* and *J*_*SC*_ as only photon absorptions in the absorber contribute to the *J*_*SC*_ of the solar cell. Thus, to optimize the bottom PSC, the thickness of the absorber layer was varied from 400 to 1000 nm by maintaining the diffusion length. Solar cell with such a low bandgap perovskite allows attaining broadband photon absorptions ranging from 300 to 1100 nm wavelength region. *QEs* of different thickness of perovskite absorber of the bottom PSC are provided in the supporting information (Fig. S11a), where corresponding *J*_SC_ ranges from 29.6 to 33 mA cm^−2^ as shown in Fig. S11b in the Supporting Information. It is seen that increasing the absorber thickness leads to enhanced *J*_SC_; however, no further improvement has been found after 800 nm, the *J*_SC_ is saturated. Typically, the bottom part of the perovskite/perovskite TSC gives a high *J*_SC_ by exhibiting a low *V*_OC_. In contrast, the top cell is responsible for enabling high *V*_OC_ with a suitable *J*_*SC*_. Hence, it is imperative to select an appropriate top cell for efficient TSC. In this study, we used the previously investigated MAPbI_3_ perovskite absorber as a top cell. By the combination of both top cell and bottom cell in a serial connection, a two-terminal (2 T) perovskite/perovskite TSC was realized, where undoped SnO_2_ is used a tunneling and interconnecting layer. The SnO_2_ layer facilitates efficient charge transportations, which can improve the *J*_SC_ and ECE of the solar cell. In TSC, the photons with high energies are absorbed by the top cell as passes low energy photons so that it can be absorbed by the bottom cell. The phenomena can be seen by the generation contour plots, as illustrated in Fig. 6, where maximum electron–hole pairs are generated in the lower bandgap materials in the solar cell structure. However, a proper realization of the matching current of the TSC is demanding while a top cell is deposited on top of a bottom cell, which needs an advanced optimization technique, including precise mesh, high-level computing, realistic design. The optimization should give an appropriate subcell thickness estimation so that the ECE is not limited by the mismatching issue. In this study, a large number of subcell combinations were selected to investigate *QE*s and *J*_*SC*_s under matching conditions. The optimization process and optics were studied by using particle swarm optimization (PSO) algorithm with 3D-FDTD optical simulations. The top cell thickness was ranging from 150 to 300 nm, while the bottom cell thickness was changing from 400 to 1000 nm. For each combination, matching *J*_*SC*_ of the perovskite/perovskite TSC is evaluated, where the contour plot, as illustrated in the supporting information (Fig. S12), demonstrates the J_SC_s for every possible combination of top and bottom cells. The matched J_SC_ is maximized while the subcell thicknesses are optimum. Findings in optical simulations reveal the upper limit of the *J*_SC_ (~ 18 mA cm^−2^) under the matching condition. The maximum matched *J*_SC_ is attained for the top and bottom cell absorber thickness combination of 210 and 800 nm, respectively. A schematic cross-section of the Perovskite/perovskite TSC under matching short-circuit current condition is depicted in Fig. 7a**,** and corresponding power density profiles under the monochromatic wavelength of 400, 550, 730, and 950 nm are presented in Fig. 7b-e. For short wavelengths (< 400 nm), most photons are absorbed by the front contact /top perovskite interface, while a fraction of the light propagates to the bulk of the perovskite absorber. The increase of wavelength (550 nm) leads to improved photon absorptions in the top absorber, and a certain portion of photons passes to the bottom cell. At 730 nm, photons are equally absorbed by both top and bottom cells, where absorption losses are pronounced in the contacts for further increase in wavelength, leading to a significant improvement of photon absorption in the bottom cell.

In contrast, almost zero absorptions appear in the top cell. Such optical phenomena and absorption mechanisms are further confirmed by the *QE* plots. Fig. 7f shows the *QE* of the best performing perovskite/perovskite TSC, which exhibits a matched *J*_*SC*_ of 18 mA cm^–2^. *QE* plot approves that photons in the shorter wavelengths mostly absorbed by the top cell, while the bottom cell absorbs most photons in the longer wavelengths. This is due to the optimum selection of the materials with suitable bandgaps. At 730 nm, both subcells work at their maximum power point; hence both top and bottom PSCs exhibit equal *QE* of ~ 45%. It is assumed that such a high *J*_*SC*_ is realized due to the efficient light incoupling and/or light trapping, which is mostly provided by the TiO_2_ ETL and front nanostructured ARC layer. The parasitic absorptions in the front are very low (< 1 mA cm^−2^); however, yet a significant portion of incident lights do not absorb in the solar cell due to higher reflections (~ 10%).

The optics of the investigated perovskite/perovskite TSC under matching condition is also supported by the electric field distribution profiles for an incident wavelength of 400, 550, 730, and 950 nm, as provided in Fig. S13. Until now, we have focused only on the optics of solar cells so that a maximum matched *J*_*SC*_ can be achieved. However, electrical effects are equally essential for determining realistic ECE of the investigated perovskite/perovskite TSC, which cannot be determined by only optical simulations. Hence, FEM calculations were used for electrical simulations of single-junction subcells and complete TSCs. Here, electron–hole pair (EHP) generations from optical simulations were used as optical input for electrical simulations. A detailed description of the TSC simulation is provided in the Methods section.

Generation rates for top single-junction PSC bottom single-junction PSC, and perovskite/perovskite are illustrated in Fig. 5a-c. As expected, a high rate of EHP generation occurred in the active layers, which are due to low material bandgaps as compared to other (contact) layers. The generation of photons decreases from top to bottom in the active layer; therefore, the generation rate is comparatively higher close to the surface. Simulations allow providing *J-V* characteristic curves of investigated solar cells, where the photovoltaic performance parameters can be extracted. The *J-V* characteristic curves of investigated individual subcells and complete TSC are shown in Fig. 7g and extracted photovoltaic parameters are listed in Table 3. The investigated single-junction top PSC exhibits an ECE of 22.7% with a *J*_SC_ of 23.6 mA cm^−2^, a *V*_OC_ of 1.15 V, and a FF of 83.5%, whereas the bottom PSC provides an ECE of 24.4% with a *J*_SC_ of 34 mA cm^−2^, a *V*_OC_ of 0.89 V, and a FF of 80.5%. By combining both single-junction PSCs in a TSC structure, the ECE is raised to 30% with a matched *J*_SC_ of 18 mA cm^−2^, a *V*_OC_ of 2.03 V, and a FF of 82.5%. Simulated electrical parameters are close to the experimentally realized values provided in literatures [53–56]. The matched *J*_SC_ from optical and electrical simulations are very much comparable, which confirms the accuracy of our provided investigations. The proposed experimental realization of device fabrication is provided in the Methods Section. As a final step, investigated photovoltaic performance parameters are compared with the theoretical upper limit (SQ limit) of solar cells so that a better understanding of the performance of the solar cell can be attained, which are demonstrated in Table 4. The SQ limits were calculated based on the original published paper by Shockley and Queisser in 1961 [13]. The SQ limit of *J*_SC_, *V*_OC_, *FF*, and ECE of the single-junction solar cells are provided in Fig. S14. It is observed that the investigated electrical parameters (*V*_OC_ and *FF*) are approaching (≥ 90%) their theoretical limit, where *J*_SC_s are ranging from 80–90% of its maximum value.

**Table 3:** Performance parameters for investigated perovskite single-junction and perovskite/perovskite tandem solar cells using FDTD optical simulation

Solar cell structure	Performance parameters						
	E_g_ (eV)	Max.GR (cm^−3^ s^−1^)	*J*_SC_ (mA cm^−2^)	*V*_OC_ (V)	FF (%)	P_max_ mW cm^−2^)	ECE (%)
Top PSC	1.6	1.2 × 10^22^	23.6	1.15	83.5	22.661	22.7
Bottom PSC	1.16	2.1 × 10^22^	34	0.89	80.5	24.356	24.4
2 T Perovskite/Perovskite TSC		1.0 × 10^22^	18	2.03	82.5	30.156	30.2

**Table 4:** Comparison of the photovoltaic performance parameters of investigated perovskite single-junction and perovskite/perovskite tandem solar cells with theoretical upper limit or Shockley-Queisser (SQ) limit

Solar cell structure	Performance parameters				
	E_g_ (eV)	*J*_SC_ / *J*_SC_^SQ^	*V*_OC_/*V*_OC_^SQ^	FF/FF^SQ^	ECE/ECE^SQ^
Top PSC	1.6	0.91	0.87	0.92	0.76
Bottom PSC	1.16	0.81	0.95	0.92	0.73
2 T Perovskite/Perovskite TSC		0.90	0.91	0.92	0.81

Nevertheless, it is assumed that the utilization of the potential surface and interface engineering can further amplify *J*_SC_s. The ECE of investigated solar cells is continuing between 70 and 80% of its theoretical value. Hence, it is believed that the provided approach has a great potential to cross the SQ limit of the single-junction solar cells and will give a pathway to realize next-generation high-efficiency solar cells. In the case of PSCs, the front contact must be efficient to provide several advantages; hence, multi-layer systems are applied. In our case, SPD-grown TiO_2_ by combined with other materials, act as the multi-layer front contact, which allows realizing high-efficiency single-junction PSCs and perovskite/perovskite TSCs. Moreover, the performance of PSCs can be further improved by exploiting efficient photonic structure, which improves the light trapping in PSCs [45, 57, 58]. Sanchez-Sobrado et al. demonstrated a low-cost light trapping structure based on TiO_2_ and IZO materials by colloidal lithography (CL) process, which could be another potential strategy for realizing an improved photovoltaic efficiency [59, 60].

## Summary

In this study, a multi-layer material system is investigated to prepare efficient front contact for perovskite single-junction and perovskite/perovskite tandem solar cells (TSCs). As a first step, a high-quality, reproducible, and scalable TiO_2_ compact electron transport layer (ETL) has been prepared by spray pyrolysis deposition (SPD), which is a vital part of the front contact. The optimization of the compact TiO_2_ has been extensively studied during the PSC fabrication. The optimized TiO_2_ ETL has a thickness of 70 nm, which exhibits a *J*_SC_ of 21.3 mA cm^−2^, *V*_OC_ of 1.07 V, *FF* of 72%, and ECE of 16.55%; up to our knowledge, this is so far the highest ECE achieved from a planar PSC with SPD-grown TiO_2_ compact ETL. In this study, rigorous 3D optical-electrical coupled electromagnetic simulations have been used to study optics and electrical characteristics of solar cells, which allows us to validate findings from the experiments further. In the next step, we have proposed a multi-layer front contact design by combining with the optimized TiO_2_ compact layer for efficient PSCs. The optimized single-junction device enhanced the ECE by 36%, with the improvement of *J*_SC_, *V*_OC_, *FF* by 10%, 7%, and 8.5%, respectively. Finally, the optimized device design has been advanced for the realization of efficient perovskite/perovskite TSCs. The PSO algorithm has carried the optimization of subcells in a tandem structure with 3D FDTD simulations. The proposed perovskite/perovskite TSC shows an ECE of ~ 30% under the matched short-circuit current condition. It is believed that such a realistic multi-layer front contact has excellent potential for the implementation of high-performance perovskite solar cells.

## Electronic supplementary material

Below is the link to the electronic supplementary material.

Supplementary file1 (PDF 2704 kb)
