# Supplementary material for: Spray Pyrolyzed TiO2 Embedded Multi-Layer Front Contact Design for High-Efficiency Perovskite Solar Cells
Source: Nanomicro Lett. 2021 Jan 4;13:36. doi: 10.1007/s40820-020-00559-2 (PMC8187539; doi:10.1007/s40820-020-00559-2)
Supplement: Supplementary file 1 — Supplementary file1 (PDF 2704 kb) [file 40820_2020_559_MOESM1_ESM.pdf]

Supporting Information for

Spray Pyrolyzed TiO_2 Embedded Multi-Layer Front Contact Design for High-Efficiency Perovskite Solar Cells

Md. Shahiduzzaman[1, 3, †, *], Mohammad Ismail Hossain[2, 8, †], Sem Visal[5], Tetsuya Kaneko[5], Wayesh Qarony[2], Shinjiro Umezu[6], Koji Tomita[4], Satoru Iwamori[3, 5], Dietmar Knipp[9], Yuen Hong Tsang[2], Md. Akhtaruzzaman[7, *], Jean-Michel Nunzi[1, 10], Tetsuya Taima[1], Masao Isomura[4, *]

[1]Nanomaterials Research Institute (NanoMaRi), Kanazawa University, Kakuma, Kanazawa 920-1192, Japan

[2]Department of Applied Physics and Materials Research Center, The Hong Kong Polytechnic University, Hung Hom, Kowloon, Hong Kong, P. R. China

[3]Research Institute of Science and Technology, and [4]Department of Chemistry, School of Science, and [5]Graduate School of Engineering, Tokai University, Kitakaname, Hiratsuka 259-1292, Japan

[6]Department of Modern Mechanical Engineering, Waseda University, 3-4-1 Okubo, Shinjuku, Tokyo 169-8555, Japan

[7]Solar Energy Research Institute, The National University of Malaysia, 43600 Bangi, Selangor, Malaysia

[8]Department of Materials Science and Engineering, City University of Hong Kong, Kowloon, Hong Kong, P. R. China

[9]Geballe Laboratory for Advanced Materials, Department of Materials Science and Engineering, Stanford University, Stanford, USA
[10]Department of Physics, Engineering Physics and Astronomy, Queens University, Kingston, Ontario, Canada
†Md. Shahiduzzaman and Mohammad Ismail Hossain contributed equally to this work

*Corresponding authors. E-mail: shahiduzzaman@se.kanazawa-u.ac.jp (Md. Shahiduzzaman); akhtar@ukm.edu.my (Md. Akhtaruzzaman); isomura@tokai.ac.jp (Masao Isomura)

S1 Supplementary Figures and Tables

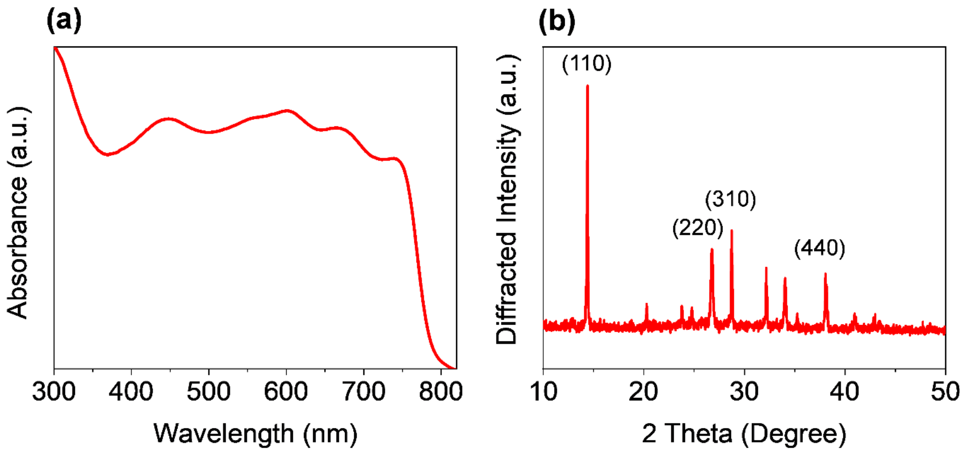

Fig. S1 a UV-vis absorption spectrum and **b** X-ray diffraction patterns of the perovskite film fabricated on the TiO_2-CL/FTO substrate. The TiO_2 precursor solution has a concentration of 0.35 M and a thickness of 70 nm

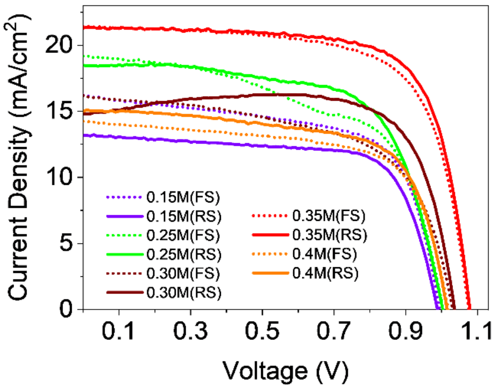

Fig. S2 *J-V* curves with the forward scan (FS) and the reverse scan (RS) of the fabricated planar PSCs for different TiO_2 precursor solution concentrations

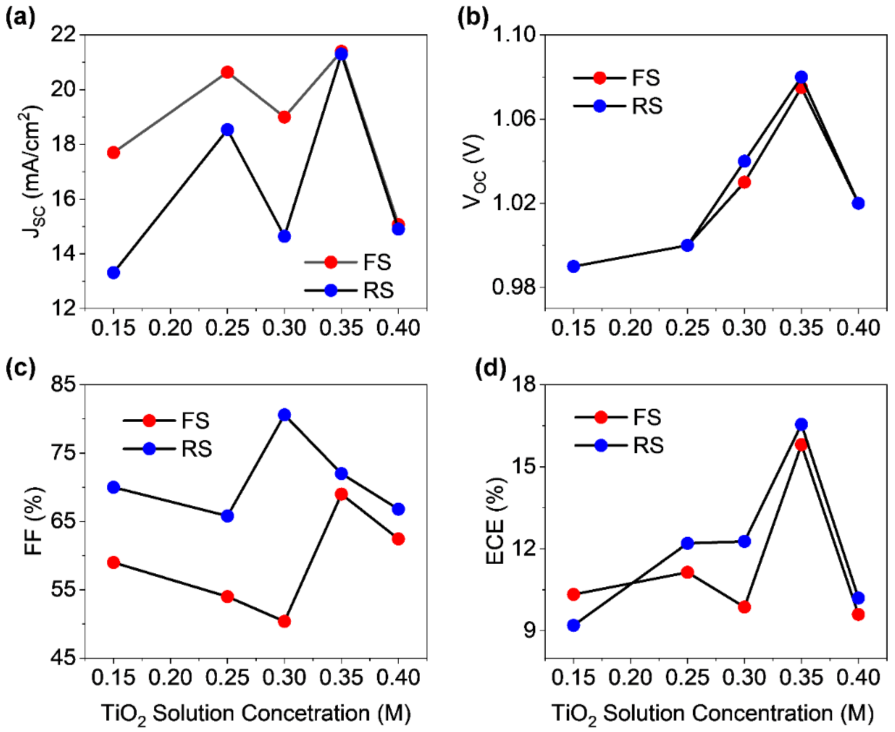

Fig. S3 TiO_2 precursor solution concentration-dependent **a** J_{SC}, **b** V_{OC}, **c** FF, and **d** ECE of fabricated devices with forward scan and reverse scan

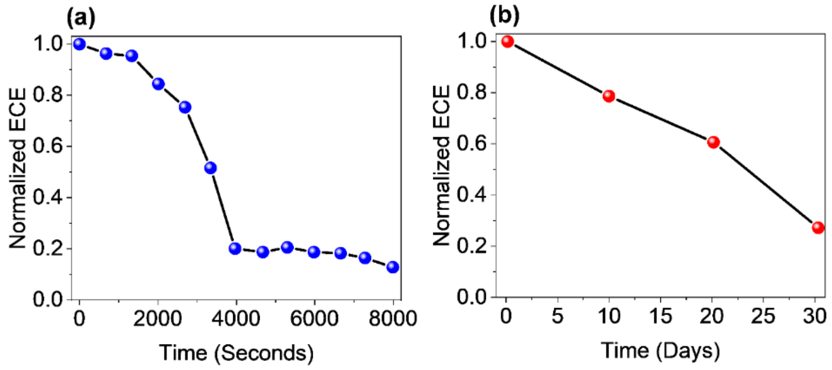

Fig. S4 a Normalized energy conversion efficiency (ECE) against time (in seconds) of planar perovskite solar cells (PSCs) integrated with only TiO_2 ETL (non-capsulated) under continuous 1 sun illumination (100 mW cm^{-2}) in air with the humidity range 60-70%. **b** Normalized ECE against time (in days) of planar PSCs integrated with only TiO_2 ETL (non-

capsulated) stored in the dark with dry N_2 in air with the humidity range 60-70%. The resultant devices were measured under the ambient 60-70% relative humidity conditions.

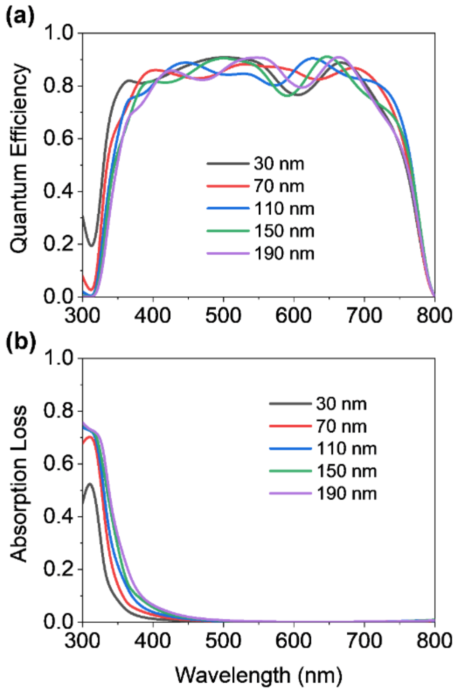

Fig. S5 A comparison of **a** quantum efficiencies and **b** parasitic absorptions by TiO_2-CL layer for different thicknesses of the TiO_2-CL film

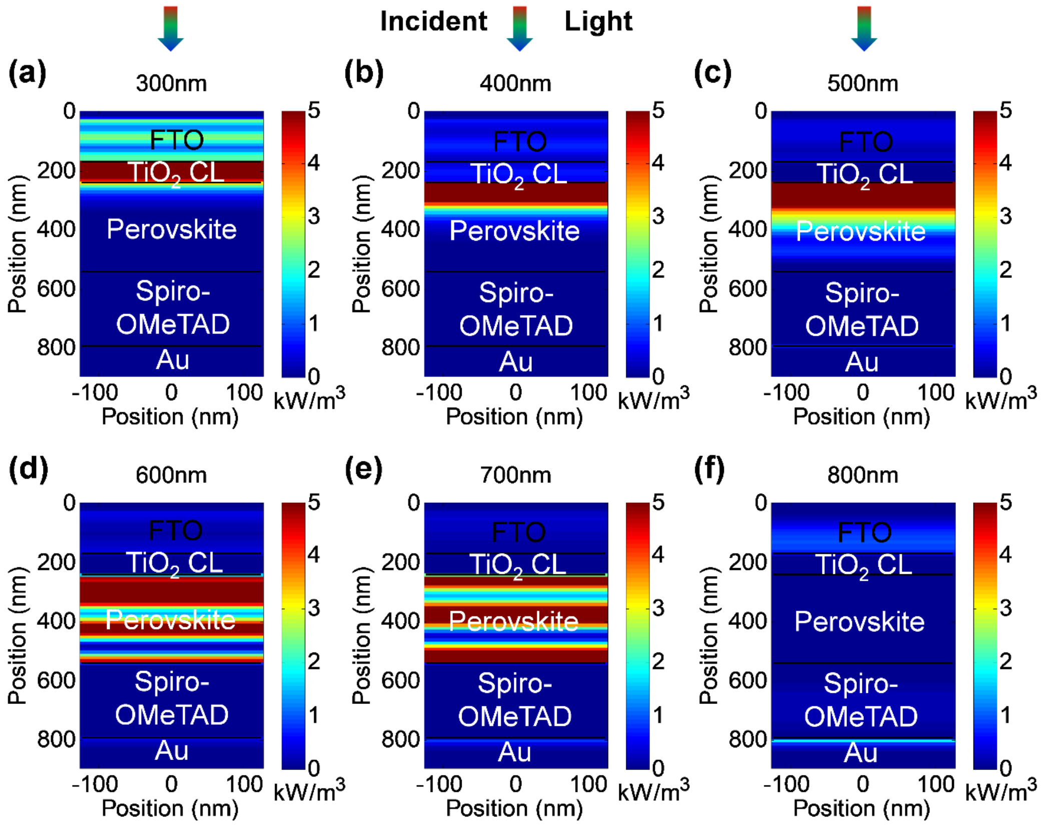

Fig. S6 Power density maps of the planar reference perovskite solar cell under monochromatic illumination of **a** 300 nm, **b** 400 nm, **c** 500 nm, **d** 600 nm, **e** 700 nm, and **f** 800 nm. The TiO_2 ETL has a thickness of 70 nm

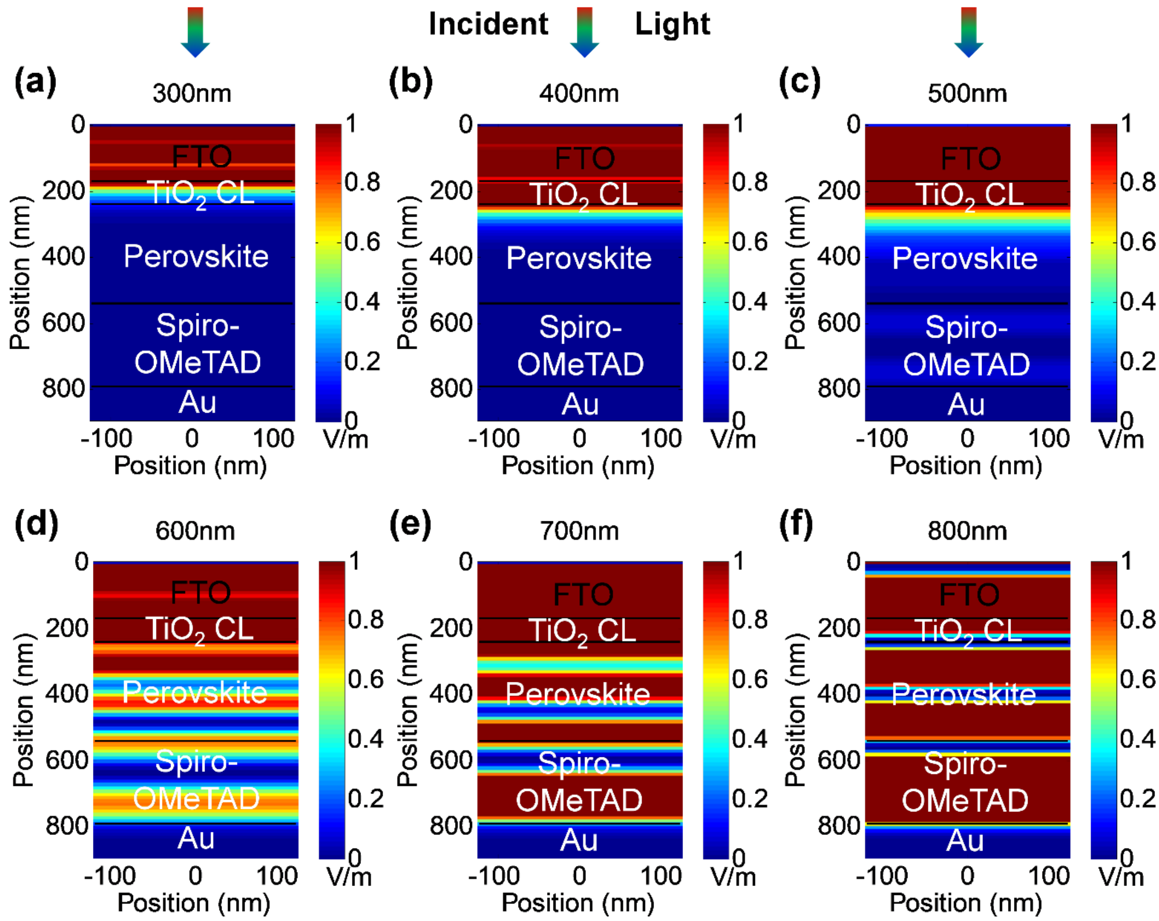

Fig. S7 Electric field distribution maps of the planar reference perovskite solar cell under monochromatic illumination of **a** 300 nm, **b** 400 nm, **c** 500 nm, **d** 600 nm, **e** 700 nm, and **f** 800 nm. The TiO_2 ETL has a thickness of 70 nm

Table S1 TiO_2 precursor solution concentration-dependent performance statistics (average ± standard deviation) of fabricated planar PSCs with a structure of FTO/TiO_2 CL/$MAPbI_3$/spiro-OMeTAD/Au

**Solution Concentration**	**Performance Parameters**			
	V_{oc} (V)	J_{sc} (mA cm^{-2})	FF (%)	ECE (%)
0.15 M	0.96±0.03	14.35±5	56±11	7.3±3
0.20 M	0.93±0.07	16.94±1.2	58±12	9.1±1.7
0.25 M	1.0±0.04	18.45±1.3	63±5	9.1±1.7
0.30 M	0.98±0.07	17.9±2.2	68±12	11.6±2.3
0.35 M	1.02±0.06	19.37±1.93	67±5	13.5±3.05
0.40 M	0.97±0.09	19.1±1.7	48±12	9.3±3.7

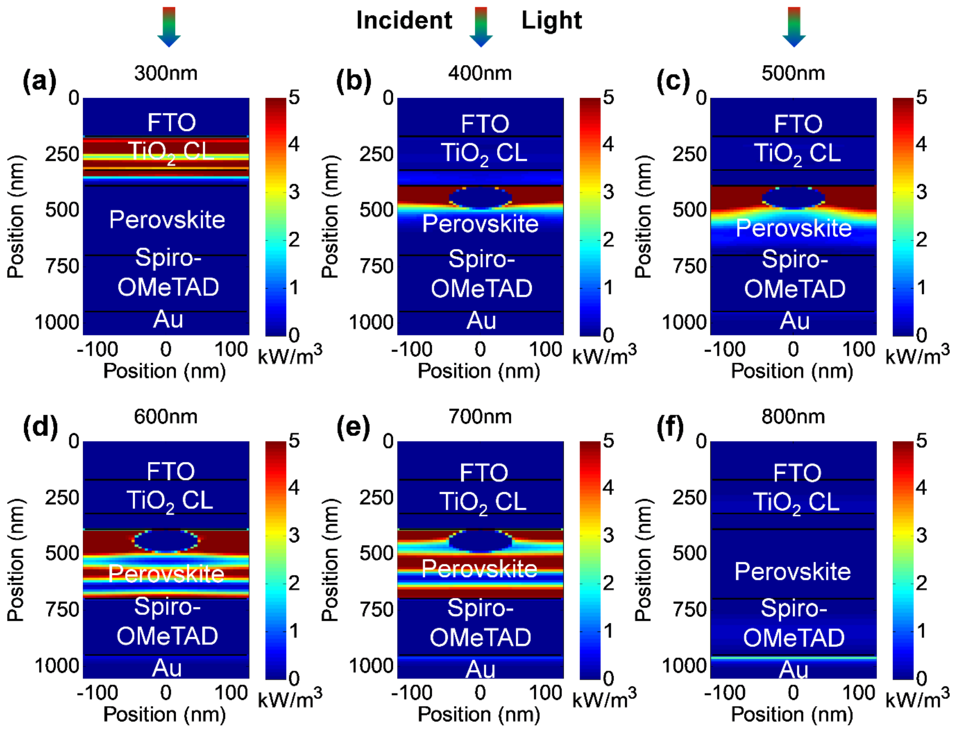

Fig. S8 Power density maps of the optimized single-junction perovskite solar cell under monochromatic illumination of **a** 300 nm, **b** 400 nm, **c** 500 nm, **d** 600 nm, **e** 700 nm, and **f** 800 nm. The TiO_2 ETL has a thickness of 70 nm, and the mp-TiO_2 nanoparticle has a diameter of 100 nm

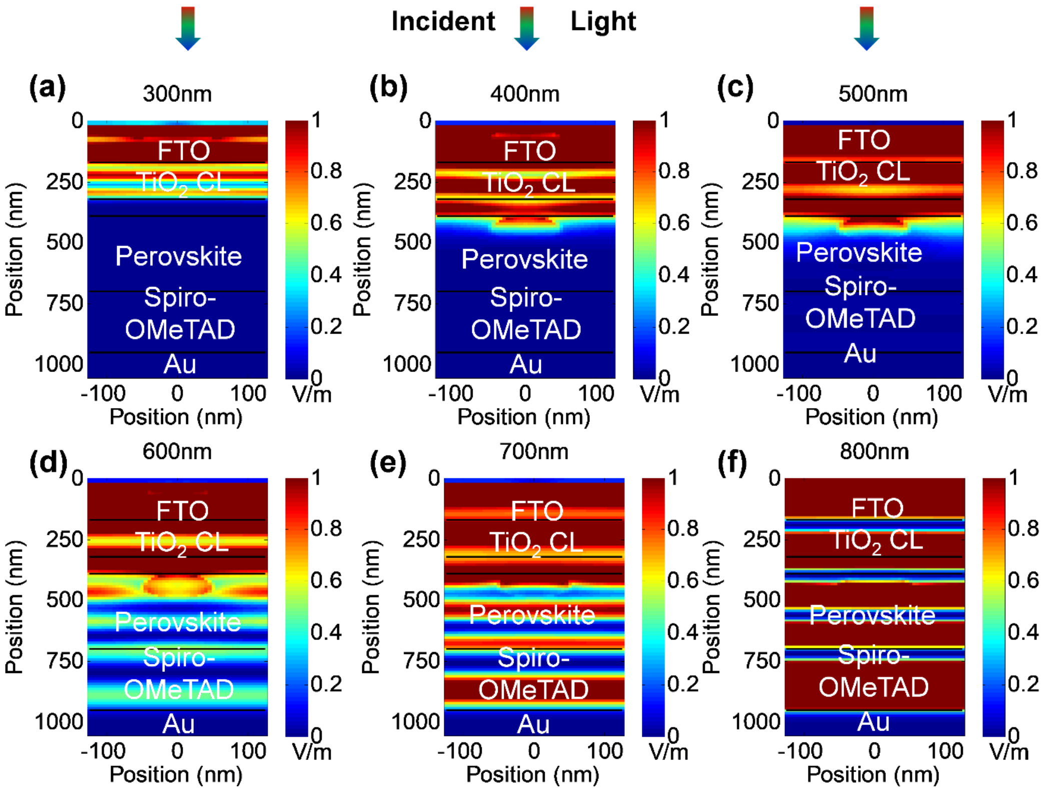

Fig. S9 Electric field distribution maps of the optimized single-junction perovskite solar cell under monochromatic illumination of **a** 300 nm, **b** 400 nm, **c** 500 nm, **d** 600 nm, **e** 700 nm, and **f** 800 nm. The TiO_2 ETL has a thickness of 70 nm, and the mp-TiO_2 nanoparticle has a diameter of 100 nm

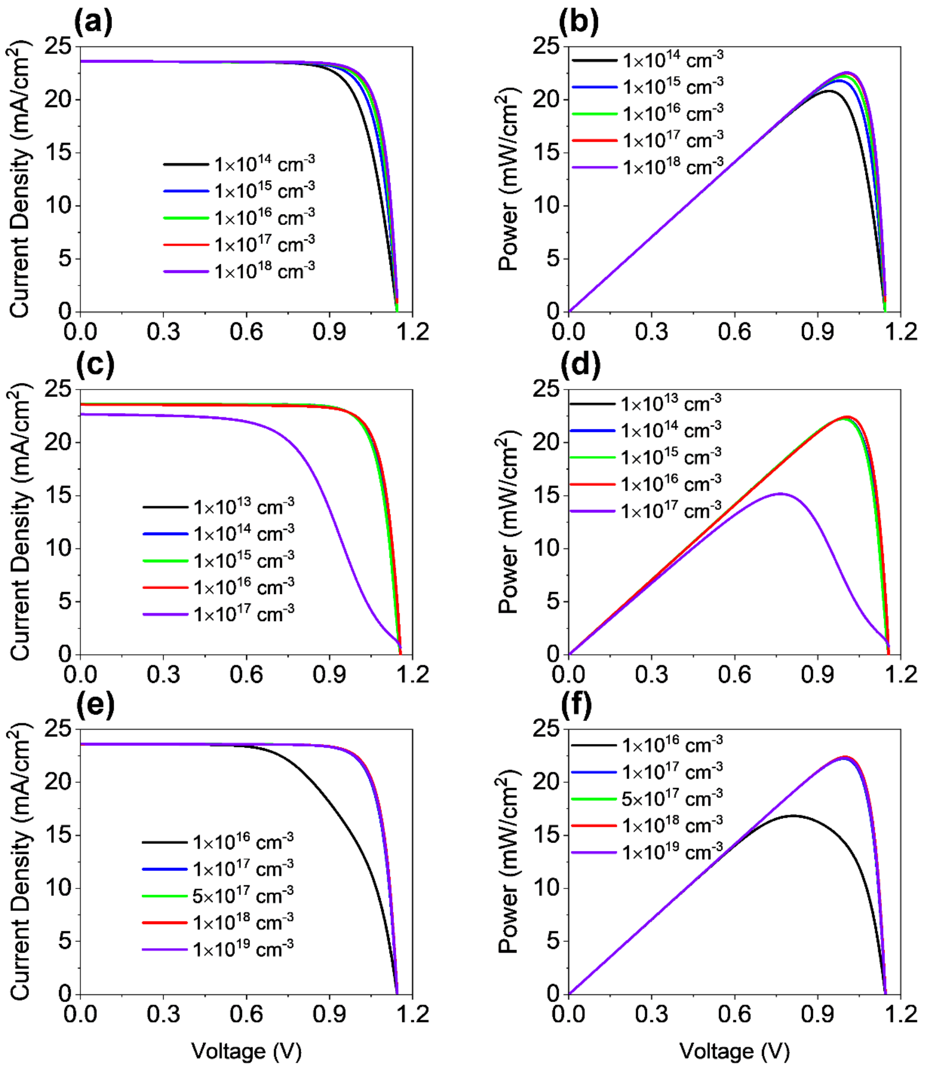

Fig. S10 Dopant concentration of the TiO_2 dependant **a** *J-V* characteristics and **b** output power of proposed single-junction PSCs. Dopant concentration of the perovskite dependant **c** *J-V* characteristics and **d** output power of proposed single-junction PSCs. Dopant concentration of the Spiro-OMeTAD dependant **e** *J-V* characteristics and **f** output power of proposed single-junction PSCs

Table S2 Influence of the TiO_2 dopant concentration on the photovoltaic performance parameters of PSCs

**Dopant Concentration**	**Performance Parameters**				
	V_{oc} *(V)*	J_{sc} *(mA cm^{-2})*	*FF (%)*	P_{max} *(mA cm^{-2})*	*ECE (%)*
1e14 cm^{-3}	1.145	23.608	77.23	20.8638	20.86
1e15 cm^{-3}	1.146	23.605	80.72	21.8303	21.83
1e16 cm^{-3}	1.149	23.607	82.76	22.4445	22.44
1e17 cm^{-3}	1.150	23.615	83.02	22.5392	22.54
1e18 cm^{-3}	1.150	23.614	83.15	22.5888	22.59

Table S3 Influence of the Perovskite dopant concentration on the photovoltaic performance parameters of PSCs

Dopant Concentration	Performance Parameters				
	V_{oc} *(V)*	J_{sc} *(mA cm^{-2})*	*FF (%)*	P_{max} *(mA cm^{-2})*	*ECE (%)*
1e13 cm^{-3}	1.1564	23.6174	81.78	22.3335	22.33
1e14 cm^{-3}	1.1564	23.6173	81.80	22.3293	22.32
1e15 cm^{-3}	1.1507	23.6206	82.09	22.3127	22.31
1e16 cm^{-3}	1.1557	23.5881	82.34	22.4478	22.45
1e17 cm^{-3}	1.219	22.6695	54.97	15.1922	15.19

Table S4 Influence of the Spiro-OMeTAD dopant concentration on the photovoltaic performance parameters of PSCs

Dopant Concentration	Performance Parameters				
	V_{oc} *(V)*	J_{sc} *(mA cm^{-2})*	*FF (%)*	P_{max} *(mA cm^{-2})*	*ECE (%)*
1e16 cm^{-3}	1.1494	23.5985	62.36	16.8498	16.85
1e17 cm^{-3}	1.1469	23.6119	82.31	22.2909	22.29
5e17 cm^{-3}	1.1476	23.6118	82.47	22.3460	22.35
1e18 cm^{-3}	1.1488	23.6069	82.76	22.4465	22.45
1e19 cm^{-3}	1.1481	23.6085	82.45	22.3526	22.35

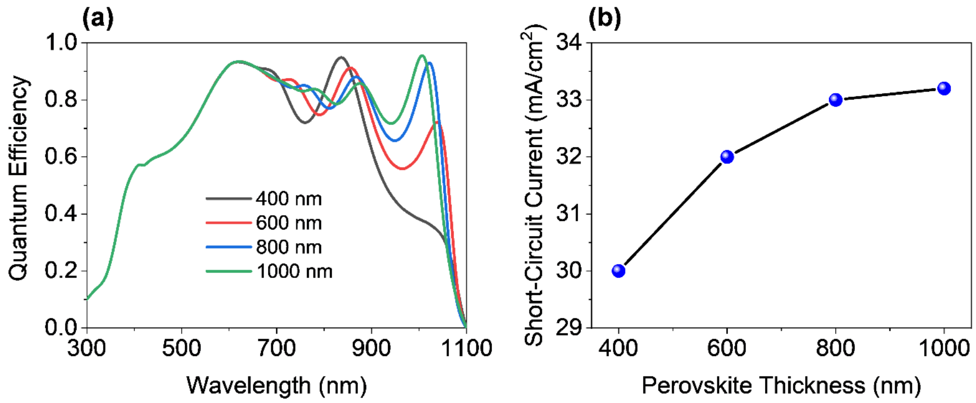

Fig. S11 A comparison of **a** quantum efficiencies and **b** short-circuit current densities of bottom perovskite solar cells for several absorber thicknesses. A $MASnPbI_3$ perovskite was used as an absorber.

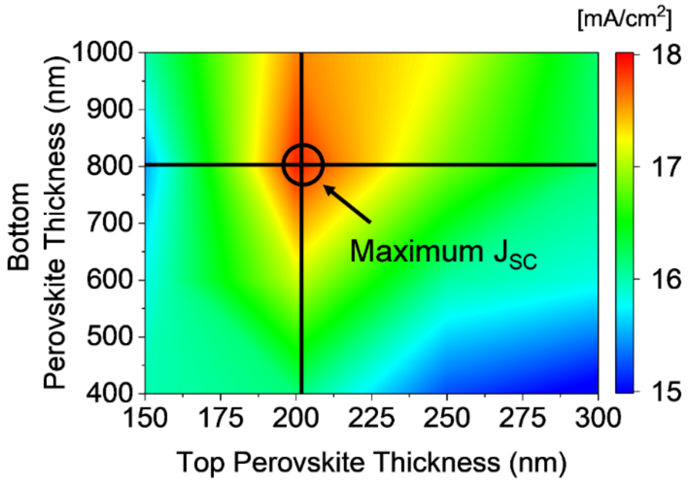

Fig. S12 Short-circuit current densities (J_{SC}s) under the matching condition of the two-terminal (2T) perovskite/perovskite tandem solar cells (TSCs) for different top and bottom cell thickness combinations

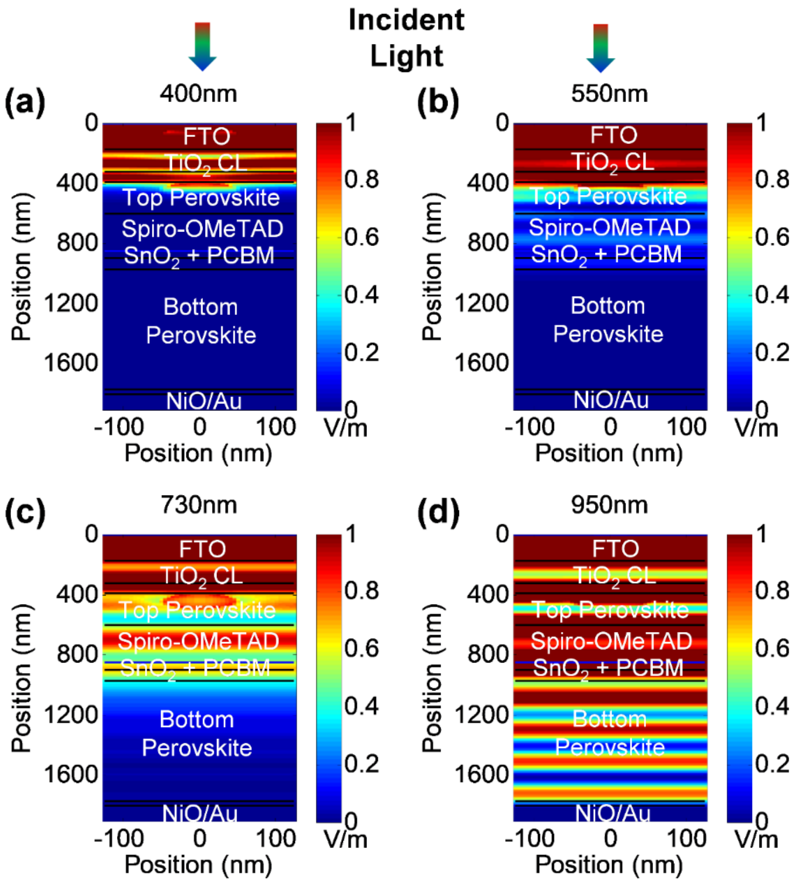

Fig. S13 Electric field distribution maps of the two-terminal (2T) perovskite/perovskite tandem solar cell under monochromatic illumination of **a** 400 nm, **b** 550 nm, **c** 730 nm, and **d** 950 nm. The top perovskite has a thickness of 210 nm, and the bottom perovskite has a thickness of 800 nm

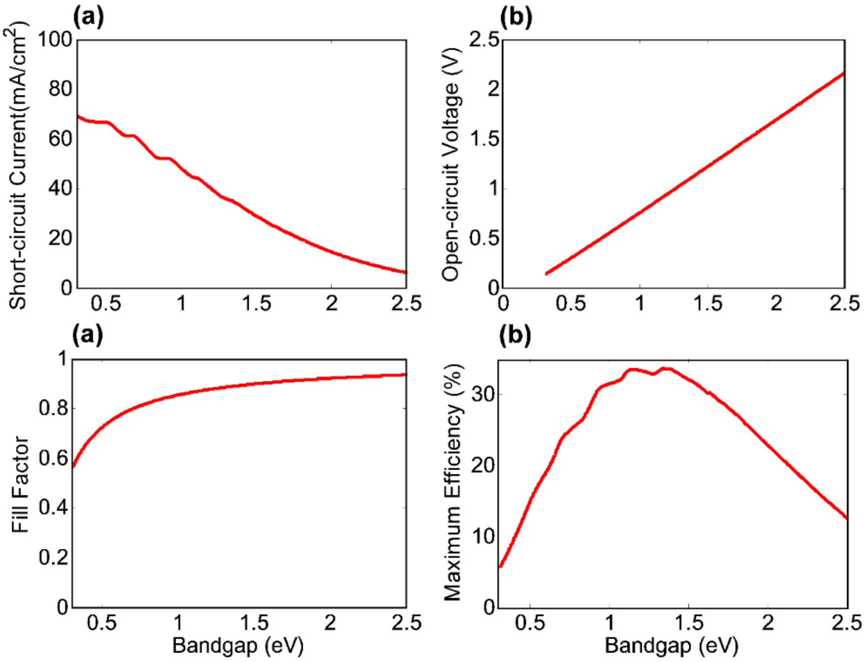

Fig. S14 Shockley-Queisser (SQ) limit for **a** short-circuit current (J_{SC}), **b** open-circuit voltage (V_{OC}), **c** fill-factor (*FF*), and **d** energy conversion efficiency (*ECE*) of the single-junction solar cell under AM 1.5G spectrum

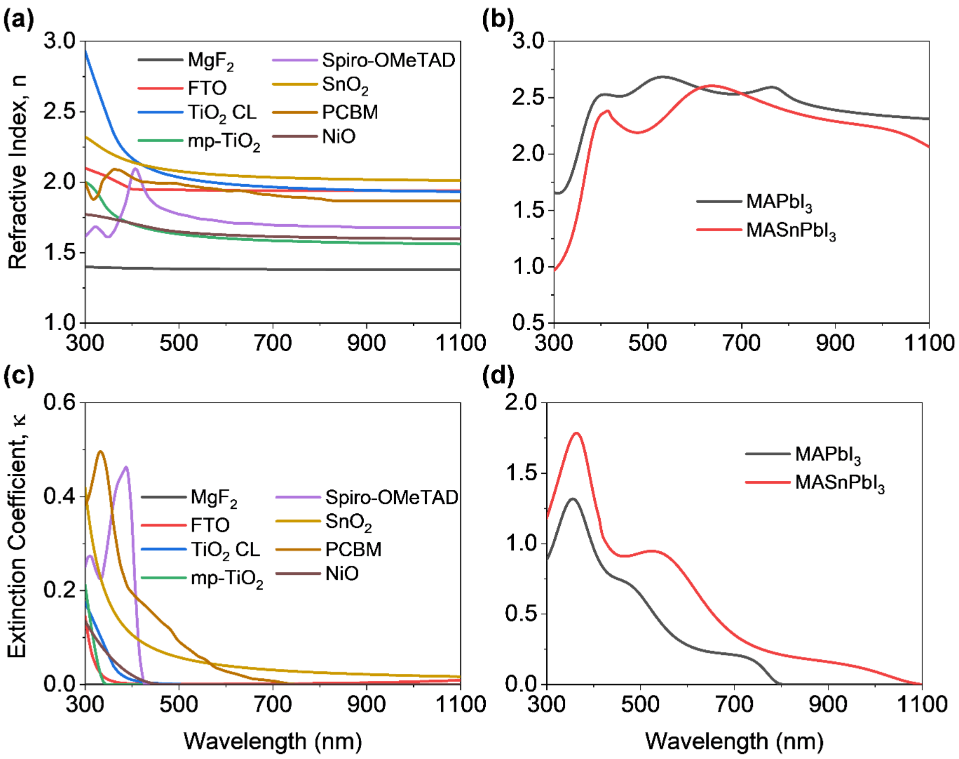

Fig. S15 a Refractive index and **c** extinction coefficient of MgF_2, FTO, TiO_2-CL, mp-TiO_2, Spiro-OMeTAD, SnO_2, PCBM, and NiO materials. **b** Refractive index and **c, d** extinction coefficient of $MAPbI_3$ and $MASnPbI_3$ perovskites.

Table S5 Necessary electrical parameters of materials used for the electrical simulations [Ref. 54-59]

**Parameters**	**Materials**						
	ETM		***Perovskite***		***HTM***		***ICL***
	TiO_2	*PCBM*	*$MAPbI_3$*	*$MASnPbI_3$*	*Spiro-OMeTAD*	*NiO*	*SnO_2*
Bandgap, E_g (eV)	3.2	2.0	1.5	1.16	3	3.6	3.5
Electron affinity, χ (eV)	4	3.9	4	4.1	2.6	1.8	4
Dielectric Permittivity, ε_r	9	4	6.5	8.2	3	11.7	9
Conduction band effective density of states, N_C (cm^{-3})	1×10^{19}	1×10^{21}	1.66×10^{19}	1×10^{18}	5×10^{19}	2.5×10^{20}	2.2×10^{18}
Valence band effective density of states, N_V (cm^{-3})	1.8×10^{16}	1×10^{20}	5.41×10^{19}	1×10^{18}	1×10^{19}	2.5×10^{20}	1.8×10^{19}
Electron mobility, μ_n ($cm^2\ V^{-1}\ s^{-1}$)	20	1×10^{-2}	50	16	2	1×10^{-3}	100
Hole mobility, μ_p ($cm^2\ V^{-1}\ s^{-1}$)	10	1×10^{-2}	50	16	5	1×10^{-3}	25
Donor density, N_D (cm^{-3})	1×10^{16}	1×10^{15}	0	3×10^{16}	0	0	1×10^{15}

Acceptor density, N_A (cm^{-3})	0	0	5×10^{13}	3×10^{16}	1×10^{18}	5×10^{17}	0
Lifetime of electron, τ_n (s)	1×10^{-5}	1×10^{-5}	1×10^{-8}	25×10^{-9}	1×10^{-6}	1×10^{-6}	1×10^{-5}
Lifetime of hole, τ_p (s)	1×10^{-6}	1×10^{-5}	5×10^{-9}	25×10^{-9}	1×10^{-5}	1×10^{-6}	1×10^{-6}
Surface recombination velocity of electrons, S_n (cm/s)	1×10^{7}	1×10^{7}	1×10^{7}	1×10^{7}	1×10^{7}	1×10^{7}	1×10^{7}
Surface recombination velocity of holes, S_p($cm\ s^{-1}$)	1×10^{7}	1×10^{7}	1×10^{7}	1×10^{7}	1×10^{7}	1×10^{7}	1×10^{7}

S2 Hybrid Approach for TSCs

In this study, we have used a hybrid technique which is applicable for both FDTD and FEM methods. More details on the hybrid approach for the realization of efficient TSCs are provided in our previous works [9, 11, 52]. In a tandem device, two complete solar cells are merged to form a single structure, where photons with high energies are absorbed by the wide bandgap top cell and allow passing low energy photons as the transmission, which are being absorbed by the low bandgap bottom cell, as depicted in Fig. S15c. In single-junction PSC, a regular solar cell structure, as shown in Fig. S15b, is considered, where perovskite absorber is sandwiched between ETL and HTL, following a metal contact at the end of the device. However, there is no metal contact in a top cell of the TSC as the top cell should transmit low energy photons to the bottom cell. Furthermore, it often requires a tunneling layer between top and bottom subcells for efficient charge carrier extraction, that contributes further to bypass electrons and holes without recombination. The top cell structure can be presented by structure A, as shown in Fig. S15a.

However, in the hybrid method, the optical wave propagation and performance analysis of the TSC can be defined by the following formula:

$$\text{QE (Top)} \approx \text{Absorption (Top Absorber)}$$

$$\text{QE (Bottom)} \approx \text{Transmission (Top Cell)} \times \text{Absorption (Bottom Absorber)}$$

Hence, it has been found that the matched J_{SC} of the TSC is constantly limited by the top cell, as Structure B (Fig. S15b) always exhibit higher J_{SC} as compared to the Structure A (Fig. S15a) due to the utilization of metal reflector that contributes to additional absorption in the active layer.

Nevertheless, for optical simulations of perovskite/perovskite TSCs, this is not a problem as the FDTD solver allows tunneling function while calculating the wave propagation. This concept greatly helps the device simulations using FEM as the device solver does not allow tunneling. Hence, we simulated two different structures (Structure A and Structure B) and optimized them for realizing TSCs with high efficiency. In a TSC, V_{OC}s of subcells are added, and minimum current from the top to bottom cells is considered a final matched J_{SC}. Moreover, the *FF* for the structure with a minimum J_{SC} is assumed to be the FF for TSCs.

A more detailed explanation is provided in our previous work on perovskite based TSCs [11].

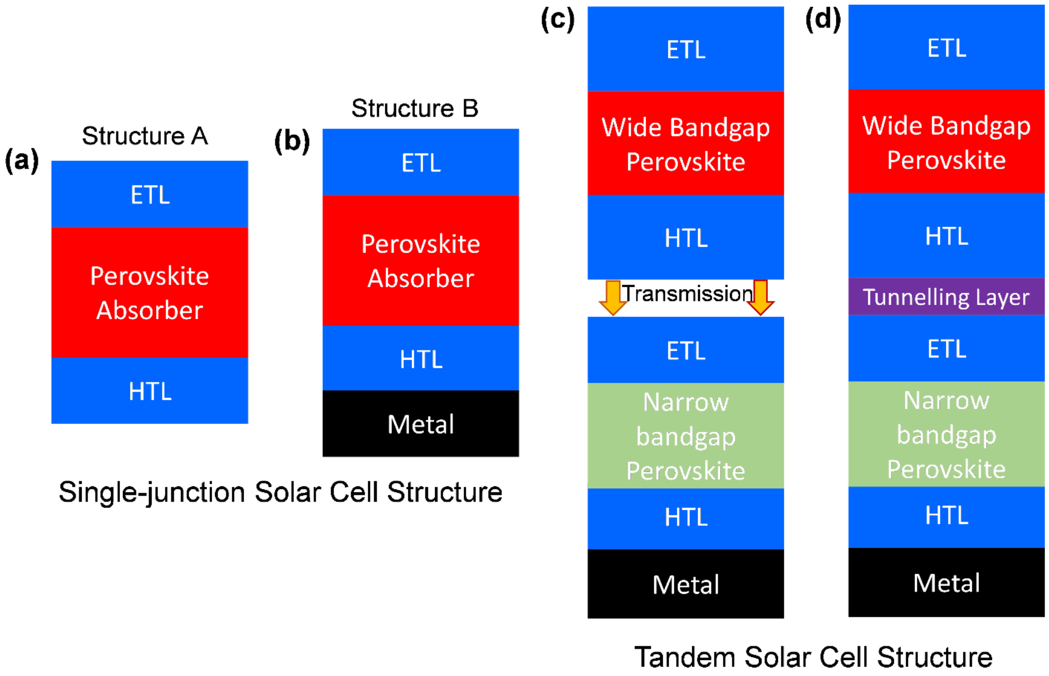

Fig. S16 Schematic cross-section of a top perovskite solar cell (PSC) **a** without and **b** with metal reflector. Schematic cross-section of the tandem solar cell (TSC) **c** without and **d** with tunneling layer

S3 Optical Simulation Method

The optical simulation allows determining electric field distributions within the simulation domain, which further let calculating the photon generation rate and power density within the solar cell. The total generation rate can be calculated by:

$$G_{Total}(\lambda) = \varepsilon'' \int \frac{|E(x,y,z)|}{2 \times h} d\lambda \qquad \text{(S1)}$$

where, ε'' is the imaginary part of the relative permittivity, $\varepsilon_r=(n-ik)^2$, where n is the refractive index and k is the extinction coefficient of the perovskite absorber. And, h is the Planck's constant. The absorption coefficient of the absorber can be computed by $\alpha=4\pi k/\lambda$. The power loss density within the solar cell can be realized by:

$$P_{Loss}(x,y,z) = \frac{1}{2} c\varepsilon_0 n\alpha |E(x,y,z)|^2 \qquad \text{(S2)}$$

where, c and ε_0 are speed of light and permittivity of free space. Calculated power densities let investigating the layer by layer loss analysis within the solar cell structure. Losses in the active or absorber layer only contributed to the Quantum Efficiency (*QE*) and J_{SC} else consider as parasitic losses. How many photons from the incident light are absorbed by the perovskite absorber can be determined by *QE*. In this study, external quantum efficiency (*EQE*) is termed as a *QE*. The *EQE* can be defined by:

$$EQE(\lambda) = [1 - R(\lambda)] \times IQE(\lambda) \qquad \text{(S3)}$$

$$QE(\lambda) \approx EQE(\lambda) \qquad \text{(S4)}$$

where, $R(\lambda)$ and $IQE(\lambda)$ the wavelength-dependent reflection and internal quantum efficiency. Hence, QE can be written as:

$$QE(\lambda) = \frac{1}{P_{incident}} \int P_{Loss}(x, y, z)\, dxdydz \tag{S5}$$

$$J_{SC} = \frac{q}{hc} \int \lambda \times QE(\lambda) \times SR_{AM1.5G}(\lambda) d\lambda \tag{S6}$$

where, q is the elementary charge. $P_{incident}$ and $SR_{AM1.5G}$ are the input optical power input and the AM 1.5G solar spectral irradiance.

$$V_{OC} = \frac{kT_{Cell}}{q} ln\left(\frac{J_{SC}}{J_0} + 1\right) \cong \frac{kT_{Cell}}{q} ln\left(\frac{J_{SC}}{J_0}\right) \tag{S7}$$

where, k is the Boltzmann constant, T_{Cell} is the temperature of the solar cell, and J_0 is the saturation current density.

S4 Electrical Simulation Method

In the FEM approach, non-linear Poisson's, continuity, and drift-diffusion equations are engaged for the calculations, which can be expressed as:

$$\nabla(\varepsilon_0 . \varepsilon_r \nabla \emptyset) = -\rho \tag{S8}$$

$$\frac{\partial_n}{\partial_t} = \frac{1}{q} \nabla j_n + G_n - R_n \tag{S9a}$$

$$\frac{\partial_p}{\partial_t} = \frac{1}{q} \nabla j_p + G_p - R_p \tag{S9b}$$

where, ε_0 and ε_r are free space permittivity and relative permittivity, respectively. Φ is defined as the electrostatic potential. G_n and G_p electron and hole generation rates, respectively, where R_n and R_p are being recombination rates for electrons and holes.

The charge (ρ) and the current density (J) can be expressed by:

$$\rho = q(n - p + N_D + N_A) \tag{S10}$$

$$J_n = -q\mu_n n \nabla \phi + qD_n \nabla n \tag{S11a}$$

$$J_p = -q\mu_p p \nabla \phi - qD_p \nabla p \tag{S11b}$$

where, n and p are electron concentration and hole concentration, respectively. N_D and N_A are donor density and acceptor density, respectively. μ_n and μ_p are electron and hole mobilities. D_n and D_p are electron and hole diffusion constants.

In this model, electron and hole generation rates were kept constant, so that the total generation rate, G_{Total}=G_n=G_p. The G_{Total} was extracted from the FDTD optical simulations, which acts as a key optical input for the electrical simulation. R is the total carrier recombination rate (R_{Total}=R_n=R_p), which can be defined by:

$$R_{Total} = R_R + R_A + R_{SRH} + R_S \tag{S12}$$

$$R_R = B(np - n_i^2) \tag{S13}$$

$$R_A = (C_n n + C_p p)(np - n_i^2) \tag{S14}$$

$$R_{SRH} = (np - n_i^2)/(\tau_p(n + n_t) + \tau_n(p + p_t)) \tag{S15}$$

$$R_S = (np - n_i^2)/\frac{1}{S_p}(n + n_{ts}) + \frac{1}{S_n}(p + p_{ts})) \tag{S16}$$

Where, R_R, R_A, R_{SRH}, and R_S are radiative, Auger, Shockley-Read-Hall, and surface recombination, respectively, where B and C_n and C_p, are radiative and Auger recombination coefficients. τ_n and τ_p are electron and hole lifetimes, where S_n and $S_p$are being surface recombination velocity of electrons and holes. And, n_i is the intrinsic carrier concentration. A set of necessary electronic parameters of materials was used for the calculation, which were adapted from the literature and summarized in Table S5. As recombination can negatively affect to the PSC mechanism; hence, in our model, we have taken into all possible recombination were considered by providing necessary values, as shown in Table S5. Besides, the Schottky contact condition was used for front contact, ohmic contact, and back contact. The sweep mesh method integrated into the electrical simulation solver was utilized to solve charge and current densities. Furthermore, a hybrid approach was used to study the electrical characteristics of TSCs, which are provided in Section S2 and Fig. S16. The close boundary conditions were applied to the simulation environment, where AZO front contact and Al metal contacts were designated as anode and cathode, respectively. For attaining the *J-V* curve, the voltage is swiped, ranging from 0 to 1.3 V for a single-junction PSC for 100 steps.

Afterward, the *ECE* of the solar cell can be calculated by:

$$ECE = \frac{P_{MPP}}{P_{incident}} = \frac{J_{SC} \times V_{OC} \times FF}{P_{incident}}$$

$$FF = \frac{P_{MPP}}{P_{incident}} = \frac{J_{MPP} \times V_{MPP}}{J_{SC} \times V_{OC}} \tag{S17}$$

where, P_{MPP} is the maximum power point of the solar cells. J_{MPP} and V_{MPP} are short-circuit current density and open-circuit voltage at the maximum power point.
